# Integrated immunoreactivity analysis of MHC-I epitopes derived from the Marburg virus glycoprotein

**DOI:** 10.3389/fimmu.2026.1902130

**Published:** 2026-10-06

**Authors:** Yulin Yang, Jing Wang, Enqi Guan, Hengzheng Wei, Yubo Sun, Jianchang Li, Yuxuan Zhang, Hao-Bo Kang, Zhenchi Fang, Jierui Zhao, Peiyuan Gao, Binjie Yao, Zhikui Li, Yongkai Wang, Yun Zhang, Chunmei Zhang, Yuanjie Sun, Rui Liu, Kun Yang, Xiyang Zhang, Dongbo T. Jiang

**Affiliations:** 1 Department of Immunology, The Key Laboratory of Bio-hazard Damage and Prevention Medicine, Basic Medicine School, Air-Force Medical University (the Fourth Military Medical University), Xi’an, Shaanxi, China; 2 Department of Rehabilitation, Tangdu Hospital, Air-Force Medical University (the Fourth Military Medical University), Xi’an, Shaanxi, China; 3 School of Biomedical Sciences, Li Ka Shing Faculty of Medicine, The University of Hong Kong, Pokfulam, Hong Kong SAR, China; 4 Military Medical Innovation Center, The Fourth Military Medical University, Xi’an, China

**Keywords:** glycoprotein, immunoreactivity, Marburg virus, MHC class I, T cell epitope

## Abstract

**Introduction:**

Marburg virus (MARV) is a hemorrhagic fever virus, and its glycoprotein (GP) represents an important target for vaccine research. MHC class I (MHC-I)-restricted T cell epitopes are critical for inducing cytotoxic CD8^+^ T cell responses. In this study, we aimed to identify MHC-I-restricted T cell epitopes derived from the MARV Angola strain GP through an integrated bioinformatics and experimental approach.

**Methods:**

We retrieved the MARV Angola strain GP sequence and divided it into 673 overlapping 9-mer peptides. By integrating five computational prediction algorithms, immunogenicity scoring tools and BLASTp conservation analysis, we screened 9-mer peptides from the MARV Angola GP. Molecular docking was performed with MHC-I structures using HPEPDOCK and molecular dynamics (MD) analysis. Bidirectional hierarchical clustering was used to analyze binding similarity across MHC-I molecules. Mutations identified in epitope regions were tested for their effect on affinity. Toxicity and allergenicity of the epitopes were predicted using dedicated tools. Finally, BALB/c and C57BL/6 mice were immunized with a DNA vaccine encoding MARV GP. IFN-γ secretion in mouse splenocytes was then measured by ELISpot, serum IgG by ELISA, and CD8^+^ T cell cytokine production by flow cytometry.

**Results:**

Among 673 GP peptides, eight epitopes (GAFFLYDRI, REGDMFPFL, DMFPFLDGL, NENDCDAEL, AGLSWIPFF, NQNNLVCRL, TGWGLGGKW, SDWGVLTNL) were predicted to exhibit high-affinity MHC-I binding, strong immunogenicity, and intraspecies conservation. Bidirectional hierarchical clustering analysis, molecular docking, sequence alignment, and toxicity/allergenicity predictions suggested binding across selected alleles, structural stability, limited impact from identified mutations, and low predicted toxicity. In subsequent ELISpot validation, eight epitopes elicited strong IFN-γ responses. In addition, serum from GP-immunized mice contained IgG that bound the corresponding linear peptide sequences.

**Conclusion:**

An integrated computational-experimental pipeline identified eight conserved, immunogenic MHC-I epitopes from MARV GP. These selected epitopes may serve as promising candidates for future vaccine design.

## Graphical Abstract

**Figure:**
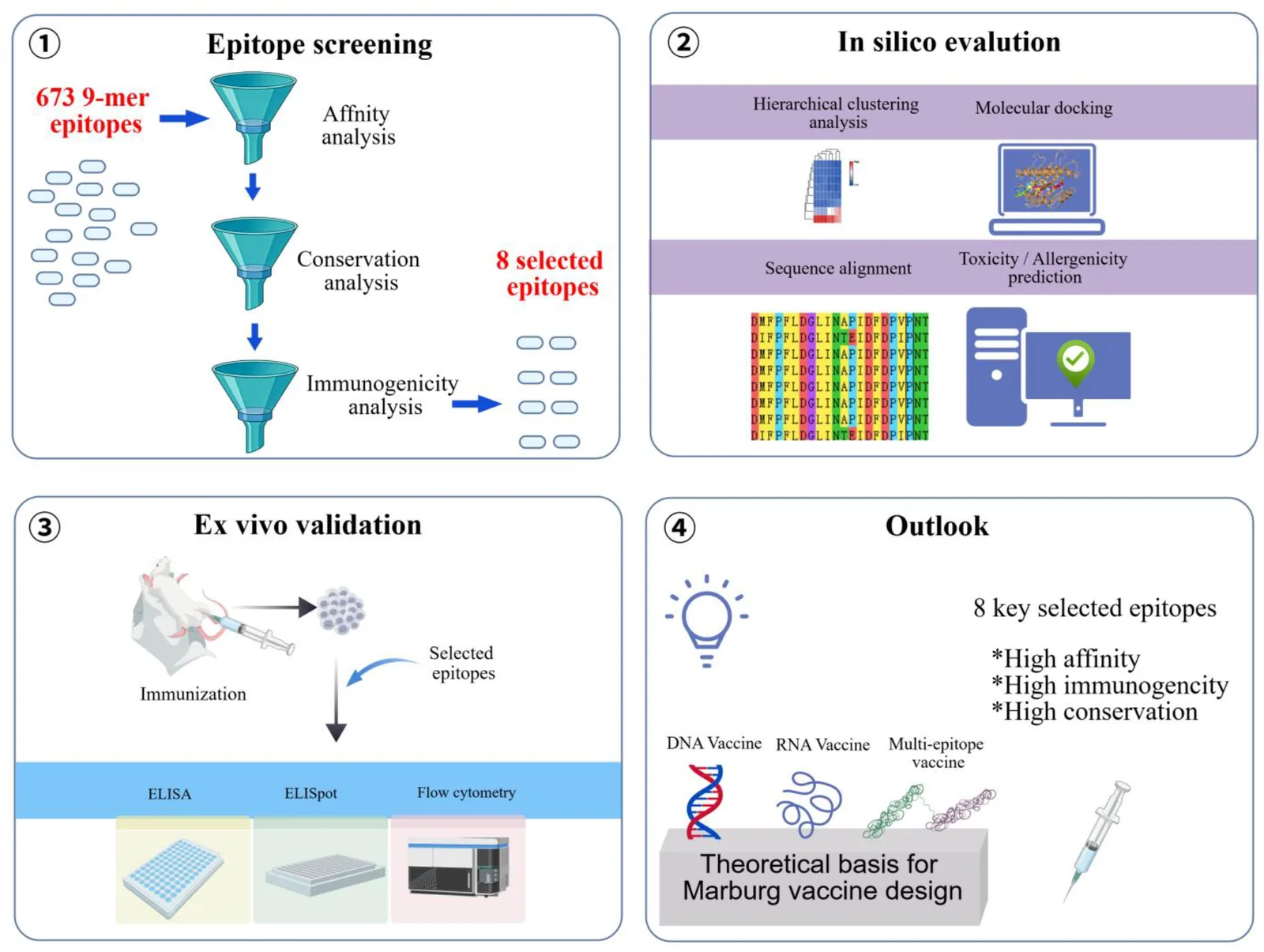
Created with BioGDP.com.

## 1 Introduction

Marburg virus (MARV) belongs to the genus Orthomarburgvirus within the family Filoviridae and the order Mononegavirales. The virus is a non-segmented, negative-sense RNA virus, with a genome of approximately 19.1 kb. The RNA encodes seven structural proteins arranged from the 3’ to 5’ end, which are NP, VP35, VP40, GP, VP30, VP24, and L (1, 2).

Transmission of the virus can occur from animal reservoirs, particularly from bats to humans. It can also occur through human-to-human transmission via sexual contact, blood, and other body fluids (3–5). Infection with MARV often presents significant therapeutic challenges and can lead to severe viral hemorrhagic fever accompanied by a robust inflammatory response. The virus is associated with a high case fatality rate (6).

During viral entry into host cells, the GP serves as a critical attachment factor. Mature MARV GP consists of GP1 and GP2 subunits, which are responsible for receptor binding and membrane fusion, respectively (7–9). The heavily glycosylated GP can influence viral tropism and facilitate immune evasion by masking antibody epitopes (10). Moreover, this immune evasion may be limited, as the glycan cap only partially shields the receptor-binding site from neutralizing nanobody binding (11, 12). Despite these challenges, GP remains a major target for vaccine development (5).

To date, no licensed vaccine or specific antiviral therapy is available for MARV infection in humans. Current research directions for MARV vaccines primarily focus on DNA vaccines and mRNA vaccines (13–15). Studies have shown that the combination of a DNA plasmid encoding GP with a recombinant adenovirus vector rAd5 can induce stronger immune responses (13). And mRNA vaccines targeting the MARV GP have also been demonstrated to elicit potent humoral and cellular immune responses in animal models (14, 15). Other antiviral therapeutic strategies have included concentrated IgG derived from convalescent patients and monoclonal antibodies (MR191-N), the latter of which can target the binding site of viral receptor (16, 17).

Immunoinformatics, defined as the systematic use of computational methods for epitope prediction, prioritization, and immune response analysis, supports vaccine design (18). Baral et al. analyzed MARV GP in terms of immunogenicity, allergenicity, IC50, and affinity. Eleven MHC-I-restricted T cell epitopes were obtained (19). Previous studies have reported the predicted MHC-I-restricted MARV GP epitopes in limited immunogenetic contexts. However, the understanding of these epitopes remains relatively limited, and further comprehensive screening is needed. Moreover, as MARV outbreaks predominantly occur in Africa with a limited number of cases, the progress of vaccine clinical trials has been slow, making it difficult to complete efficacy validation within outbreak windows. Although Ebola virus and MARV both belong to the family Filoviridae, they lack cross-protection, further underscoring the urgency of developing specific MARV vaccines.

The Angola strain, responsible for the largest and most lethal Marburg virus disease outbreak on record (2004–2005 in Uíge Province, Angola), exhibits stronger pathogenicity than other reported strains with a case fatality rate of 90%. In animal experiments, rhesus macaques infected with Angola Marburg virus showed more rapid disease progression compared to those infected with other strains (6, 10, 20). Furthermore, compared with MARV Musoke GP, the steric shielding effect of Angola Marburg virus GP was more significant (20). Therefore, we focused on the GP of Lake Victoria marburgvirus Angola2005 as the study target, and performed a multidimensional predictive analysis to identify MHC-I-restricted T cell epitopes with high affinity, strong immunogenicity, and high conservation.

## 2 Materials and methods

### 2.1 MARV GP sequences retrieval

The amino acid sequence of MARV GP (Accession No: Q1PD50) was obtained from NCBI GenBank. The strain selected in this experiment was Lake Victoria marburgvirus Angola2005. To investigate the effects of amino acid mutation in GP, we also obtained GP mutant strains within the genus Orthomarburgvirus from NCBI GenBank (94 envelope GP sequences in **Supplementary Table 1**).

### 2.2 Population coverage

To comprehensively predict the binding of the epitopes of MARV GP to HLA-I, we first identified the preferred HLA-I molecules for each epitope based on the predicted binding affinity. These preferred HLA-I alleles were then used as input for the IEDB Population Coverage tool to evaluate the theoretical population coverage across major geographical regions (21, 22).

### 2.3 MARV GP MHC-I epitopes prediction and screening

To comprehensively obtain candidate epitopes with high-affinity, we selected five online tools with different algorithms (23–27) (All the database versions and web servers used in the study are shown in **Supplementary Table 2**). Bioinformatics tools for affinity screening were included in databases IEDB, SMMPMBEC from the IEDB, NetMHCpan4.1, SYFPEITHI and Rankpep. (A) IEDB: Combines artificial neural network, stabilized matrix method (SMM) and other algorithms into a consensus score to predict MHC-epitope binding affinity with reduced bias and increased robustness (23). (B) SMMPMBEC: Uses a novel amino acid similarity matrix (PMBEC) derived from combinatorial peptide library binding data as a Bayesian prior within the SMM to predict MHC-epitope binding affinity (25). (C) NetMHCpan4.1: Predicts MHC-epitope binding affinity by integrating binding affinity data and mass spectrometry-eluted ligands through a tailored machine learning strategy (24). (D) SYFPEITHI: Predicts MHC-epitope binding affinity by scoring each amino acid position based on a motif-derived matrix that assigns higher values to anchor and preferred residues observed in naturally processed MHC ligands, then summing the scores to rank candidate peptides (26). (E) Rankpep: RANKPEP predicts MHC-epitope binding affinity by deriving a position-specific scoring matrix (PSSM) from a set of aligned peptides known to bind a given MHC molecule, and then ranking all candidate peptides from a protein based on their similarity to this PSSM (27).

The rationale for employing all five tools is that each algorithm is based on a different principle (consensus, matrix, neural network, motif-based, and PSSM). By combining their predictions, we can identify candidate epitopes that are consistently ranked highly across multiple methods. Peptides that appear most frequently among the top-scoring candidates from all five tools are selected as the most reliable high-affinity epitopes.

The 681-amino acid GP was scanned with a sliding 9-mer window, generating 673 overlapping 9-mer peptides. To facilitate subsequent validation in a mouse model and to identify epitopes with potential cross-species immunogenicity, we selected six H-2 molecules of mice and nine HLA-I superfamily molecules. The H-2 molecules were H-2-D^b^, H-2-D^d^, H-2-K^b^, H-2-K^d^, H-2-K^k^ and H-2-L^d^. The nine HLA-I superfamily molecules are listed in **Supplementary Table 3** (28).

In each algorithm, the binding affinity between different epitopes and each MHC-I molecule was ranked from high to low. The top 2% of combinations within each algorithm were defined as high-affinity peptides. To identify MHC-I peptides with high-affinity fairly and comprehensively, we listed the peptides with high-affinity obtained by each algorithm. Peptides were retained if they appeared in at least three of the five algorithms. For the peptides not covered by all five algorithms, those ranked in the high-affinity subset in at least two algorithms were retained. The top 2% cutoff was selected to balance sensitivity and specificity, consistent with the consensus prediction strategy established in a landmark immunoinformatics study (29).

### 2.4 Immunogenicity analysis

Immunogenicity reflects the ability of antigens to activate T cells. Studies showed that epitopes with high MHC-I binding affinity did not necessarily have strong immunogenicity (30). To select epitopes with strong immunogenicity to CD8^+^ T cells, we used two algorithms IEDB and PAAQD 2.0 to predict all the antigenic epitopes (31). The final epitopes with strong immunogenicity were defined as the intersection of the predictions from both algorithms.

For the results obtained by the IEDB algorithm, we considered epitopes with an immunogenicity score > 0 as having strong immunogenicity. For the results obtained from the PAAQD 2.0 algorithm, we considered epitopes with an immunogenicity score > 0.5 as having strong immunogenicity. The immunogenicity cutoffs were applied as follows: For IEDB, we followed the recommended threshold (>0) (32). For PAAQD 2.0, we used the default classification boundary of the random forest classifier implemented in the PAAQD 2.0 tool, where peptides with a predicted probability > 0.5 are assigned as immunogenic (Positive) (33).

### 2.5 Conservation analysis

To characterize viral-sequence conservation of the dominant epitopes, we used BLASTp to assess the conservation of MARV GP epitopes. We evaluated the intraspecific conservation and intergeneric conservation. If the evaluation result E-value less than or equal to 1×10^-5^, we considered the epitope conserved. Otherwise, it was considered non-conserved. An E-value ≤ 1×10^-5^ is a widely accepted BLASTp threshold for defining conserved sequences (34). Because an E-value for a 9-mer is influenced by database size and does not directly represent an exact-match conservation frequency, we interpret this analysis as similarity-based conservation screening rather than a direct measure of population frequency.

Intraspecific conservation was assessed against other strains of Orthomarburgvirus marburgense (taxid: 3052505), excluding the reference strain Lake Victoria marburgvirus-Angola2005 (taxid: 378830). The same E-value threshold (≤ 1×10^-5^) was applied to the intraspecific conservation evaluation, consistent with the intergeneric analysis and without any alteration to the cutoff. Intergeneric conservation was assessed against other members of the family Filoviridae (taxid: 11266), excluding the species Orthomarburgvirus marburgense (taxid: 3052505).

### 2.6 Molecular docking of selected epitopes to MHC-I structures

We used MHC-I molecules and Angola MARV GP epitopes to simulate molecular docking. After screening for affinity, immunogenicity, and conservation, selected epitopes were submitted to docking to better predict their binding strength. We obtained PDB files from the RCSB PDB database and used the HPEPDOCK 2.0 server to simulate the molecular docking (35). To achieve broader coverage of HLA-I molecules and to better evaluate the binding affinity between dominant epitopes and these molecules, we obtained a total of 13 MHC-I molecule structures from the RCSB PDB database (**Supplementary Table 4**).

The interaction between the MHC-I molecule and the dominant antigen epitope displayed distinct binding modes, each with a corresponding docking score. Each epitope bound to each MHC-I molecule in up to 100 different binding modes. The top ten binding modes and the corresponding docking scores were selected for further evaluation. To quantitatively assess the molecular docking results, we analyzed the best docking score and number of favorable poses. Because HPEPDOCK reports docking scores as negative values, a more negative score indicates stronger predicted binding. The best docking score was defined as the most negative docking score (the largest absolute value of the docking score). The larger the absolute value of the docking score was, the stronger the binding force. The best docking score for each epitope-MHC pair was normalized using Z-score transformation. We took the median of all the normalized scores as a threshold. For each group, the number of poses with an absolute normalized score above this threshold was defined as number of favorable poses. A bubble chart was then generated using RStudio to visualize the processed data.

The static 3D models generated by molecular docking cannot capture the dynamic behavior of binding between the same MHC molecule and distinct dominant epitopes. For epitope-MHC complexes with favorable docking scores, we performed MD simulations to evaluate the stability of the binding modes obtained from docking. During the prespecified analysis window, structural descriptors included RMSD, RMSF, principal component analysis (PCA), and radius of gyration (Rg). Peptide-chain and whole-complex RMSD metrics were explicitly distinguished wherever both are reported. These MD analyses were used as descriptive structural characterization and are not interpreted as direct evidence of immunogenicity or protective efficacy (36).

MD simulations were performed using NAMD 3.0.2 (multicore-CUDA) with the CHARMM36 force field. Each docked complex was placed in a rectangular box with a minimum solute-box distance of 2.4 Å, solvated with TIP3P water, and neutralized/ionized with 0.15 M NaCl. After energy minimization for 10,000 steps using NAMD’s minimize algorithm, the system was under NPT conditions during the production run (5 ns production run) at 300 K and 1.01325 bar using a Langevin thermostat with damping coefficient of 1 ps^-1^ and a Langevin piston with period of 100 fs and decay of 50 fs. Production trajectories were run for 5 ns with a 2.0-fs time step, saving coordinates every 5,000 steps (10 ps). One independent trajectory was generated per complex. All frames from the entire production trajectory (0–5 ns) were included in the analysis. Prior to analysis, the production trajectory was superimposed onto the first frame by least-squares fitting of the protein Cα atoms. Structural metrics (RMSD, RMSF, radius of gyration) and a principal component analysis (PCA) were then computed on the fitted trajectories using MD analysis.

### 2.7 MARV GP epitopes and MHC-I clustering

The polymorphism of MHC-I molecules and the diversity of antigenic epitopes contribute to the variability in immune responses. To better visualize the results and characterize the similarity between different MHC-I molecules, we performed bidirectional hierarchical clustering analysis. A heat map was drawn using TBtools (37). Affinity data were processed by 1- $log_{2}^{rank}$(rank value is affinity ranking) to optimize visualization clarity. The affinity data were derived from IEDB analysis of 673 peptides binding to 31 selected HLA-I and H-2 molecules. The high-affinity was represented in red and the low affinity was represented in blue.

### 2.8 Sequence alignment of MARV variants

After identifying epitopes with high-affinity, strong immunogenicity and high conservation, we further analyzed all GP mutant strains within the species. Based on the Lake Victoria Marburg virus Angola2005 strain, a total of 94 GP sequences from mutant strains were selected. The 94 GP sequences were aligned by MEGA11 software (38). Then we submitted the alignment results to WebLogo (39). The WebLogo output clearly revealed the types of amino acid variations at each site. This allowed us to clearly identify whether the dominant epitopes from the Lake Victoria marburgvirus-Angola 2005 strain harbored any mutations in the reported strains.

For each dominant epitope protein sequence, we identified the types of mutations present and calculated the mutation probabilities. The mutation sites with high probability were selected for further analysis, and the impact of the amino acid mutations on the surrounding continuous epitope sequence was reassessed. The affinity was evaluated by the IEDB algorithm and the rank scores were processed in a 1- $log_{2}^{rank}$. The larger result after processing indicated the higher affinity. Finally, TBtools was used to draw the heat maps to assess the mutation results. For each single amino acid mutation, this approach visualized the differences in affinity between the mutant sequence and the original sequence across different MHC-I molecules.

To better illustrate the impact of an amino acid mutation on the affinity between the surrounding epitope sequence and the MHC-I molecules, we used Origin 2024 software to generate the scatter plots before and after the mutation.

### 2.9 Prediction of epitope toxicity and allergenicity

When the DNA vaccine entered the host cells, the host cells synthesized GP, which stimulated the production of corresponding antibodies. However, the epitopes synthesized by the DNA vaccine may exhibit toxicity or allergenicity. Therefore, it was critical to predict the toxicity and allergenicity of the dominant epitopes. In this experiment, the DNA vaccine was designed to synthesize MARV GP. ToxinPred 3.0 was an algorithm to predict the toxicity of epitopes, when the prediction score was greater than 0.8, it was considered indicative of toxicity (40). AlgPred 2.0 was an algorithm for epitope allergenicity prediction. The prediction score was greater than 0.4, indicating potential allergenicity (41).

### 2.10 Vaccine, animals, immunization, and euthanasia

The DNA vaccine used in this experiment was synthesized using the pVAX1 vector by Wuhan GeneCreate Biological Engineering Co., Ltd, and cloned into plasmids. The plasmids we used were isolated and purified using the Plasmid Maxi Kit (DP117; TIANGEN, Beijing, China). The vaccine was generated in a single batch, and was verified to be free of mutations by gene sequencing. The GP encoding DNA was derived from MARV Angola. Healthy 8-week-old female BALB/c and C57BL/6 mice (n=12 each group), were purchased from the Animal Experimental Center of PLA Air Force Medical University. The mice were housed in a sterile environment, where controlled conditions (temperature: ~27 °C, humidity: ~50%) and a 12/12h light/dark cycle were maintained. They had free access to sterilized drinking water and standard food pellets. For each mouse strain, six mice were administered the DNA vaccine every three weeks for a total of two inoculations of 30 µL (1 µg/µL). The remaining six mice per strain served as the negative control group and received an equivalent volume of sterile PBS. All mice were euthanized two weeks after the last inoculation and splenocytes were collected. These two inbred strains were selected for two key reasons. First, their stable genetic background and defined MHC haplotypes improve reproducibility for peptide-response screening. Second, our *in silico* clustering of predicted peptide-binding profiles showed partial overlap between selected murine H-2 molecules and human HLA-I supertypes, providing a pragmatic basis for comparing murine and human binding patterns. This overlap does not imply immunogenetic equivalence between mouse H-2 and human HLA-I, and translation of individual epitopes to humans requires HLA-restricted validation.

All animal experiments followed the “3R” (Reduction, Replacement, Refinement) principle. During the whole process of animal experiments, mice were treated humanely and experiments were carried out according to relevant ethical standards. Euthanasia was performed in accordance with the American Veterinary Medical Association (AVMA) Guidelines for the Euthanasia of Animals (2020). The animals were placed in a CO_2_ chamber starting from room air (approximately 0.04% CO_2_) that had not been pre-filled with CO_2_. Medical-grade CO_2_ was then introduced at a rate of 30% of the chamber’s volume per minute, gradually raising the internal CO_2_ level to above 70%, which led to rapid deep anesthesia and death with minimal suffering. After breathing stopped, CO_2_ delivery continued for at least another minute. Immediately following this, cervical dislocation was performed. Death was then confirmed by checking for the absence of respiration and the loss of all vital signs. All efforts were made to minimize animal suffering, and no surgical procedures were performed in this study.

### 2.11 ELISpot assay

Peptides with high-affinity, strong immunogenicity and high conservation were synthesized (Shanghai Apeptide Co., Ltd., Shanghai, China). The IFN-γ capture antibody (BD Pharmingen) was diluted 1:200 in buffer solution to achieve a final concentration of 5 μg/mL. Then, 100 μL of the diluted antibody solution was added to each well of the ELISpot plate, and the plate was incubated overnight at 4°C.

The next day, the antibody was removed, and the wells were washed once with RPMI 1640 medium containing 10% FBS (both from Corning Inc., Corning), then blocked with the same medium for 2 hours at room temperature. The mice were euthanized, and their spleens were collected to prepare single cell suspensions. Red blood cell lysis buffer was added to remove erythrocytes. After lysis, the splenocytes were washed and resuspended in RPMI 1640 medium with 10% FBS (both from Corning Inc., Corning) and counted. The blocking solution (Corning Inc) was removed and 2×10^6^ lymphocytes were added to each well. The synthetic GP polypeptide sequences were diluted to a concentration of 5 μg/mL and added 100μL to the well as a stimulant. The plate was incubated in a CO_2_ incubator at 37°C for 24 hours. RPMI 1640 medium with 10% FBS was used as a negative control and concanavalin A (5 μg/mL) as a stimulator was used as a positive control.

On the third day, lymphocytes were removed and ELISpot plates were washed with water followed by PBST. Then, 100 μL of 2 μg/mL biotinylated anti-mouse IFN-γ antibody was incubated for 2 hours. The antibody was then removed and the plate was washed four times with PBST. 100 μL of streptavidin-HRP (BD Pharmingen) was added to each well and incubated for 1 hour at room temperature. HRP was removed after 1 hour and the ELISpot plate was washed with water. 100 μL of AEC substrate solution (Beijing Solarbio Science & Technology co., Ltd.) was added, and the reaction developed until clear spots appeared. Finally, the plates were air-dried and the CTL ELISpot Reader was used to count. To minimize the potential errors, automated counts were verified by manual counting. Each experiment was repeated three times. For ELISpot, six biological replicates (mice) per strain were used. Each biological sample was assessed in duplicate wells (technical replicates). The experimental results were expressed as the average number of spots produced by 10^6^ splenocytes.

### 2.12 Enzyme-linked immunosorbent assay

MARV GP antibodies in mouse serum were detected via ELISA. Purified MARV GP peptides at a concentration of 5 mg/mL were diluted with coating buffer to 10 μg/mL, and 100 μL per well was added to 96-well ELISA plates and incubated overnight at 4 °C. Each well was blocked with PBS containing 2% BSA for 2 hours at 37 °C, followed by four washes with PBST (PBS containing 0.05% Tween-20). Immunized mouse sera were diluted 1:150 in PBST containing 0.1% BSA, and 100 μL of the diluted serum was added to each well and incubated for 1.5 hours at 37 °C. After serum removal, the plates were washed six times with PBST and twice with PBS. Subsequently, HRP-conjugated goat anti-mouse IgG (Proteintech, Wuhan, China) diluted 1:10000 was added and incubated for 1 hour at room temperature. After removing the secondary antibody, the plates were washed six times with PBST and twice with PBS. Freshly prepared TMB substrate solution (ensuring that hydrogen peroxide was not inactivated) was added at 100 μL per well and incubated in the dark at room temperature for 15–30 minutes. Finally, 50 μL of ELISA stop solution was added to terminate the reaction, and the absorbance was measured at 450 nm using a standard microplate reader. Excess immunized mouse serum was used as a positive control, while PBS containing 1% BSA served as the negative control. Each experiment was repeated three times. For ELISA, six biological replicates (mice) per strain were used, and each serum sample was measured in duplicate wells (technical replicates).

### 2.13 Flow cytometry

The method for obtaining mouse splenocytes was the same as that used in the ELISpot assay. For flow cytometry, splenocytes from the six immunized BALB/c mice were selected as biological replicates. BALB/c mice’s splenocytes were selected as the experimental material for flow cytometry in this study. The cells were divided into the following two groups: the control group consisted of splenocytes from mice immunized with the DNA-GP vaccine and cultured with the cell stimulation mixture (Invitrogen; Thermo Fisher Scientific, Inc.) only. The experimental group comprised splenocytes from mice immunized with the DNA-GP vaccine. These splenocytes were then stimulated with both the cell stimulation mixture and the GP peptide pool (a mixture of 8 GP peptides diluted in PBS, at a total peptide concentration of 20 µg/mL with each peptide at a final concentration of 2.5 µg/mL). For intracellular cytokine staining, the splenocytes were first stimulated for 4–6 hours at 37 °C, then processed with Cytofix/Cytoperm solution (BD Pharmingen). After fixation and permeabilization, splenocytes were washed once with PBS and twice with flow cytometry staining buffer containing 2% BSA. After washing, the lymphocytes were resuspended and stained with fluorochrome-conjugated monoclonal antibodies diluted in flow cytometry staining buffer for 30 minutes at 4 °C in the dark. The following antibodies (BioLegend, San Diego, CA, USA) were used for cytokine detection: CD3-FITC, CD8-Pacific Blue, and IL2-APC or IFN-γ-APC. The cells were then washed again with flow cytometry staining buffer and resuspended in 300 µL of the same buffer. Detection was performed using a NovoCyte Flow Cytometer (Agilent Technologies, Inc.) with NovoExpress 1.6.2 acquisition software (ACEA Biosciences, Hangzhou, China). For data analysis, sequential gating was performed to identify CD8^+^ T cells. First, single splenocytes were selected using FSC-A and FSC-H parameters, followed by gating on CD8^+^ T cells using a Pacific Blue-conjugated anti-CD8 antibody.

### 2.14 Statistical analysis

All statistical analyses were performed using GraphPad Prism version 9.5 (GraphPad Software, La Jolla, CA, USA). Data were expressed as mean ± SD unless otherwise indicated. For ELISpot and ELISA experiments, data were analyzed by two-way ANOVA with mouse strain (BALB/c vs. C57BL/6) and peptide epitope as independent factors. For flow cytometry experiments, the normality of the paired differences was verified using the Shapiro-Wilk test. A two-tailed paired t test was used to compare the experimental group with the control group, as each experimental-control pair was derived from the same cell preparation batch. Pairing effectiveness was evaluated using Pearson correlation coefficients. Significance levels are indicated as *P < 0.05, **P < 0.01, ***P < 0.001, ****P < 0.0001.

## 3 Results

### 3.1 Affinity analysis identified 221 GP epitopes for human HLA-I molecules or mouse H-2 molecules

Following *in silico* prediction and data processing, a total of 221 9-mer epitopes were identified as having high-affinity for either human HLA-I molecules or mouse H-2 molecules from the initial 673 9-mer peptides. Among these, 204 9-mer epitopes exhibited high-affinity for HLA-I molecules, and 82 9-mer epitopes exhibited high-affinity for H-2 molecules. Sixty-five epitopes exhibited high affinity for both HLA-I and H-2 molecules. **Tables 1**, **2** summarize human epitopes with high-affinity to HLA-I superfamily molecules and mouse epitopes with high-affinity to H-2 molecules.

**Table 1:** Number of 9-mer peptides in Marburg GP with high affinity to human HLA-I molecules.

MHC-I haplotypes	Prediction tools	GP peptides	GP (short-listed)
HLA-A1	IEDB	91	65
	NetMHCpan	68	
	Rankpep	0	
	SMMPMBEC	28	
	SYFPEITHI	0	
HLA-A2	IEDB	64	47
	NetMHCpan	49	
	Rankpep	16	
	SMMPMBEC	13	
	SYFPEITHI	14	
HLA-A3	IEDB	69	57
	NetMHCpan	62	
	Rankpep	3	
	SMMPMBEC	31	
	SYFPEITHI	23	
HLA-A24	IEDB	54	14
	NetMHCpan	35	
	Rankpep	0	
	SMMPMBEC	12	
	SYFPEITHI	18	
HLA-B7	IEDB	87	45
	NetMHCpan	62	
	Rankpep	3	
	SMMPMBEC	31	
	SYFPEITHI	45	
HLA-B8	IEDB	38	25
	NetMHCpan	29	
	Rankpep	0	
	SMMPMBEC	7	
	SYFPEITHI	0	
HLA-B15	IEDB	38	22
	NetMHCpan	29	
	Rankpep	0	
	SMMPMBEC	7	
	SYFPEITHI	0	
HLA-B44	IEDB	30	31
	NetMHCpan	21	
	Rankpep	0	
	SMMPMBEC	7	
	SYFPEITHI	15	
HLA-B58	IEDB	36	27
	NetMHCpan	24	
	Rankpep	0	
	SMMPMBEC	12	
	SYFPEITHI	161	

* GP peptides refer to the peptides with rank values in the top 2% of each individual algorithm. GP (Short-Listed) refers to GP peptides that appear at least three times when all five algorithms have results, or GP peptides that occur at least twice in the case of four algorithms or three algorithms with results.

**Table 2:** Number of 9-mer peptides in Marburg GP with high affinity to mouse H-2 molecules.

MHC-I haplotypes	Prediction tools	GP peptides	GP (short-listed)
H-2-D^b^	IEDB	35	16
	NetMHCpan	28	
	Rankpep	4	
	SMMPMBEC	16	
	SYFPEITHI	18	
H-2-Dd^d^	IEDB	40	25
	NetMHCpan	27	
	Rankpep	0	
	SMMPMBEC	2	
	SYFPEITHI	0	
H-2-K^b^	IEDB	27	17
	NetMHCpan	17	
	Rankpep	0	
	SMMPMBEC	7	
	SYFPEITHI	0	
H-2-K^d^	IEDB	32	10
	NetMHCpan	27	
	Rankpep	2	
	SMMPMBEC	7	
	SYFPEITHI	18	
H-2-K^k^	IEDB	38	29
	NetMHCpan	28	
	Rankpep	0	
	SMMPMBEC	13	
	SYFPEITHI	26	
H-2-L^d^	IEDB	33	24
	NetMHCpan	23	
	Rankpep	0	
	SMMPMBEC	8	
	SYFPEITHI	14	

*GP peptides refer to the peptides with rank values in the top 2% of each individual algorithm. GP (Short-Listed) refers to GP peptides that appear at least three times when all five algorithms have results, or GP peptides that occur at least twice in the case of four algorithms or three algorithms with results.

### 3.2 Immunogenicity of MARV GP epitopes

To effectively elicit an immune response, an epitope must exhibit not only a high binding affinity but also strong immunogenicity. In the IEDB algorithm, we identified 108 epitopes with strong immunogenicity. In PAAQD 2.0 algorithm, we identified 292 epitopes with strong immunogenicity. The intersection of the results from the two methods identified 49 epitopes with strong immunogenicity. Among these 49 epitopes, further intersection with high-affinity epitopes for HLA-I molecules identified 47 epitopes that are both strongly immunogenic and high-affinity for HLA-I. Intersecting the same 49 epitopes with high-affinity epitopes for H-2 molecules identified 16 epitopes exhibiting both high-affinity and strong immunogenicity for H-2 molecules. In total, 14 epitopes were identified as having high-affinity binding to both HLA-I and H-2 molecules as well as strong immunogenicity. The immunogenicity results are summarized in **Table 3**.

**Table 3:** The high-affinity epitopes in immunogenicity screening.

MHC-I haplotypes	Probable antigen (number)	Probable non-antigen (number)
H-2-D^b^	2	14
H-2-D^d^	3	22
H-2-K^b^	4	13
H-2-K^d^	1	9
H-2-K^k^	7	22
H-2-L^d^	5	19
HLA-A1	18	47
HLA-A2	9	38
HLA-A3	12	45
HLA-A24	5	9
HLA-B7	8	37
HLA-B8	5	20
HLA-B15	7	15
HLA-B44	8	23
HLA-B58	8	19

### 3.3 Sequence conservation of MARV GP dominant epitopes

We identified epitopes characterized by high-affinity for both HLA-I molecules and H-2 molecules and strong immunogenicity, and then assessed their intraspecific and intergeneric conservation. Three candidates (CICRIFTKY, KWWTSDWGV, and ILWREGDMF) met both the intraspecific and intergeneric sequence conservation criteria and showed high predicted affinity for human HLA-I molecules. However, because they did not meet the high-affinity threshold for murine H-2 molecules, they were not included in the shared HLA-I/H-2 epitope set used for mouse validation. No epitope simultaneously met both the intraspecific and intergeneric sequence-conservation criteria and showed high predicted binding affinity for both human HLA-I and murine H-2 molecules. Then we focused our analysis on intraspecific sequence conservation. This analysis identified 9 conserved dominant epitopes for mouse H-2 and 25 for human HLA molecules, among which 8 showed high affinities to both HLA and H-2, as well as strong immunogenicity and sequence conservation. These eight epitopes exhibited high-affinity, strong immunogenicity, and high sequence conservation, which were designated as selected epitopes. The detailed information of these eight epitopes is shown in **Table 4**. No epitope exhibited both intraspecific and intergeneric sequence conservation in humans or mice. The genus Orthomarburgvirus contains only a single species. Accordingly, we categorized the selected epitopes based on intraspecific sequence conservation status. The result is shown in the **Table 5**.

**Table 4:** The specific data of selected epitopes.

Position	Selected epitopes	MHC-I alleles			
141	GAFFLYDRI	H-2-K^b^	HLA-B*51:01	H-2-D^b^	
440	REGDMFPFL	HLA-B*40:01	HLA-B*44:03	H-2-K^K^	
		HLA-B*44:02	H-2-L^d^		
443	DMFPFLDGL	H-2-K^b^	HLA-A*68:02	HLA-A*26:01	HLA-A*02:01
		HLA-B*51:01	HLA-A*02:06		
508	NENDCDAEL	HLA-B*40:01	H-2-K^k^	HLA-B*44:03	HLA-B*44:02
528	AGLSWIPFF	H-2-D^d^	HLA-A*32:01	H-2-K^b^	HLA-B*58:01
		HLA-A*23:01	HLA-B*57:01	H-2-L^d^	HLA-A*24:02
		HLA-A*30:02	H-2-D^b^		
551	NQNNLVCRL	H-2-K^k^	HLA-A*02:06	HLA-B*40:01	
636	TGWGLGGKW	HLA-B*58:01	HLA-B*57:01	HLA-B*53:01	H-2-D^d^
		HLA-A*32:01			
647	SDWGVLTNL	H-2-K^k^	HLA-B*40:01	H-2-L^d^	H-2-K^b^
		HLA-B*44:03	H-2-K^d^		

**Table 5:** Conservation of the dominant epitopes in Marburg GP.

MHC-I haplotypes	Intraspecies+	Intraspecies-
H-2-Db	1	1
H-2-Dd	2	2
H-2-Kb	3	1
H-2-Kd	0	0
H-2-Kk	5	5
H-2-Ld	3	2
HLA-A1	16	12
HLA-A2	4	11
HLA-A3	8	11
HLA-A24	11	8
HLA-B7	5	7
HLA-B8	2	3
HLA-B15	5	2
HLA-B44	7	10
HLA-B58	10	5

### 3.4 Analysis of population coverage of selected epitopes

To achieve comprehensive coverage of the predominant MHC-I molecules within the human population, a total of 27 HLA-I alleles were selected for epitope prediction analysis. These alleles collectively cover 98.34% of the human population. Geographically, the predictive analyses were primarily concentrated in the Americas, Europe, and Asia, with a balanced distribution among the three regions. In addition, studies from several other areas were also included, collectively forming a broad geographical coverage. The global population coverage estimates for the selected epitopes are summarized in **Table 6**. Among individual epitopes, DMFPFLDGL exhibited the highest coverage (Global: 50.86%, East Asian: 53.94%, European: 58.27%, North American: 55.56%), followed by AGLSWIPFF (Global: 37.09%). In contrast, GAFFLYDRI (7.43%) and NQNNLVCRL (9.61%) showed relatively limited coverage when used alone. The combined coverage of all eight epitopes reached 80.39% globally, with particularly high coverage in East Asia (87.93%), North America (86.54%), and Europe (83.37%). However, coverage in South America (63.19%), South Asia (62.91%), and Northeast Asia (64.08%) was comparatively lower, suggesting the need for additional epitopes tailored to these regions. Importantly, the African population, where MARV outbreaks predominantly occur, achieved a combined coverage of 73.58%, indicating that the current epitope panel provides substantial but not complete coverage.

**Table 6:** Global population coverage of selected epitopes.

Population	Estimated coverage by GAFFLYDRI	Estimated coverage by REGDMFPFL	Estimated coverage by DMFPFLDGL	Estimated coverage by NENDCDAEL	Estimated coverage by AGLSWIPFF	Estimated coverage by NQNNLVCRL	Estimated coverage by TGWGLGGKW	Estimated coverage by SDWGVLTNL
World	7.43%	20.88%	50.86%	20.88%	37.09%	9.61%	13.86%	13.83%
East Asian	15.07%	21.11%	53.94%	21.11%	52.49%	21.6%	4.64%	20.2%
Northeast Asian	7.68%	16.97%	30.0%	16.97%	33.18%	19.51%	10.73%	16.16%
South Asian	8.15%	7.01%	31.96%	7.01%	36.46%	9.04%	20.96%	5.54%
European	9.7%	26.38%	58.27%	26.38%	32.97%	7.48%	13.7%	14.62%
South American	3.96%	10.34%	32.11%	10.34%	33.03%	3.29%	5.26%	6.99%
North American	8.37%	24.34%	55.56%	24.34%	42.24%	11.18%	17.73%	17.94%
Oceanian	1.17%	16.59%	18.91%	16.59%	56.14%	13.96%	4.59%	14.21%
African	5.56%	10.0%	32.82%	10.0%	44.0%	0.9%	23.6%	8.5%

### 3.5 Bidirectional hierarchical clustering reflects MHC-I binding profiles between MHC-I molecules and MARV GP

We next performed a bidirectional hierarchical cluster analysis to compare predicted peptide-binding profiles across MHC-I molecules. The analysis used all MARV GP 673 peptides, and a heat map was generated with TBtools based on the affinity data obtained from IEDB. The heat map was shown in **Supplementary Figure 1**.

The 31 selected HLA-I and H-2 molecules were classified into three groups: HLA-I exclusive, H-2 exclusive, and cross-reactive clusters (HLA-I major). In the cross-reactive clusters, several murine H-2 molecules showed predicted binding-profile similarities to selected HLA-I supertypes. The affinity of H-2-K^k^ to each epitope was similar to that of the HLA-B44 (HLA-B*40:01, HLA-B*44:02, HLA-B*44:03). Similarly, the affinity of H-2-K^d^ was similar to HLA-A24 (HLA-A*24:02, HLA-A*23:01). The affinity of H-2-L^d^ for each epitope was comparable to those of HLA-B7 and HLA-B8. These observations indicate that the predicted binding repertoires of certain H-2 molecules overlap with those of human HLA-I alleles. This supports the use of this mouse model in subsequent experiments. Likewise, proximity among HLA-I clusters reflects similarity in predicted peptide-binding profiles and does not by itself establish population protection. In our clustering results, the affinity of HLA-B15 (HLA-B*15:01) to each epitope was similar to that of HLA-A1 (HLA-A*01:01, HLA-A*26:01, HLA-A*30:01).

### 3.6 Molecular docking suggests stable binding between selected epitopes and MHC-I molecules

We next assessed the binding of the eight selected epitopes to different MHC-I molecules using molecular docking simulations (**Figure 1**). The results revealed that the selected epitopes TGWGLGGKW, AGLSWIPFF, GAFFLYDRI and REGDMFPFL exhibited favorable docking scores across the H-2 alleles and HLA-I subtypes. This indicated a potential high degree of interaction with these MHC-I molecules. The selected epitopes SDWGVLTNL, DMFPFLDGL and TGWGLGGKW had a large number of favorable poses with H-2 alleles and HLA-I subtypes, indicating a high probability of stable binding. The specific data of molecular docking is shown in **Supplementary Table 5**. These results were visualized in **Figure 2**.

**Figure 1:**
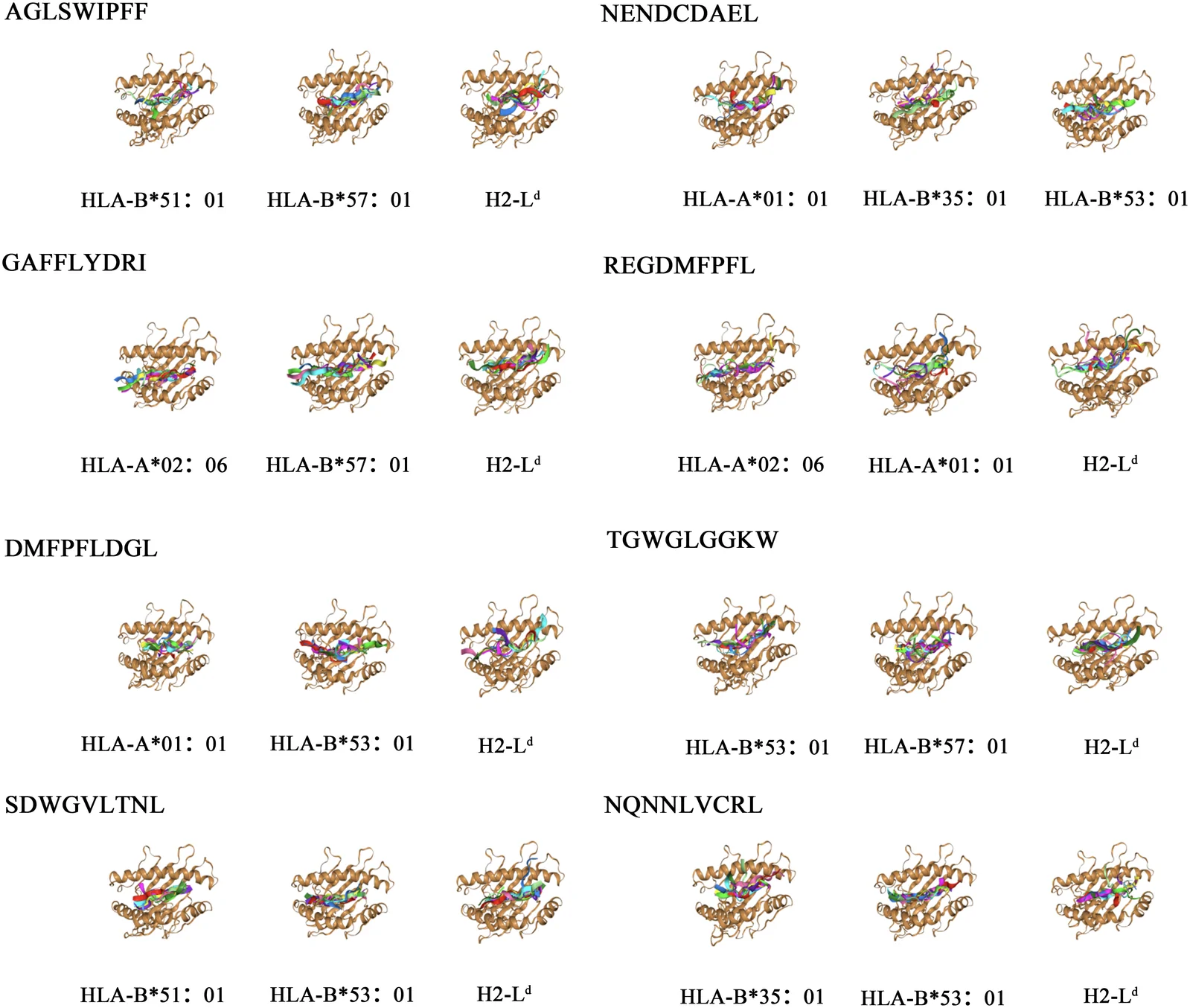
Docking models of eight selected epitopes with their corresponding MHC-I alleles. For each epitope, docking models are arranged from left to right in descending order of the absolute values of their docking scores, with a larger absolute value indicating stronger binding affinity. This *in silico* analysis should not be interpreted as direct evidence of immunogenicity.

**Figure 2:**
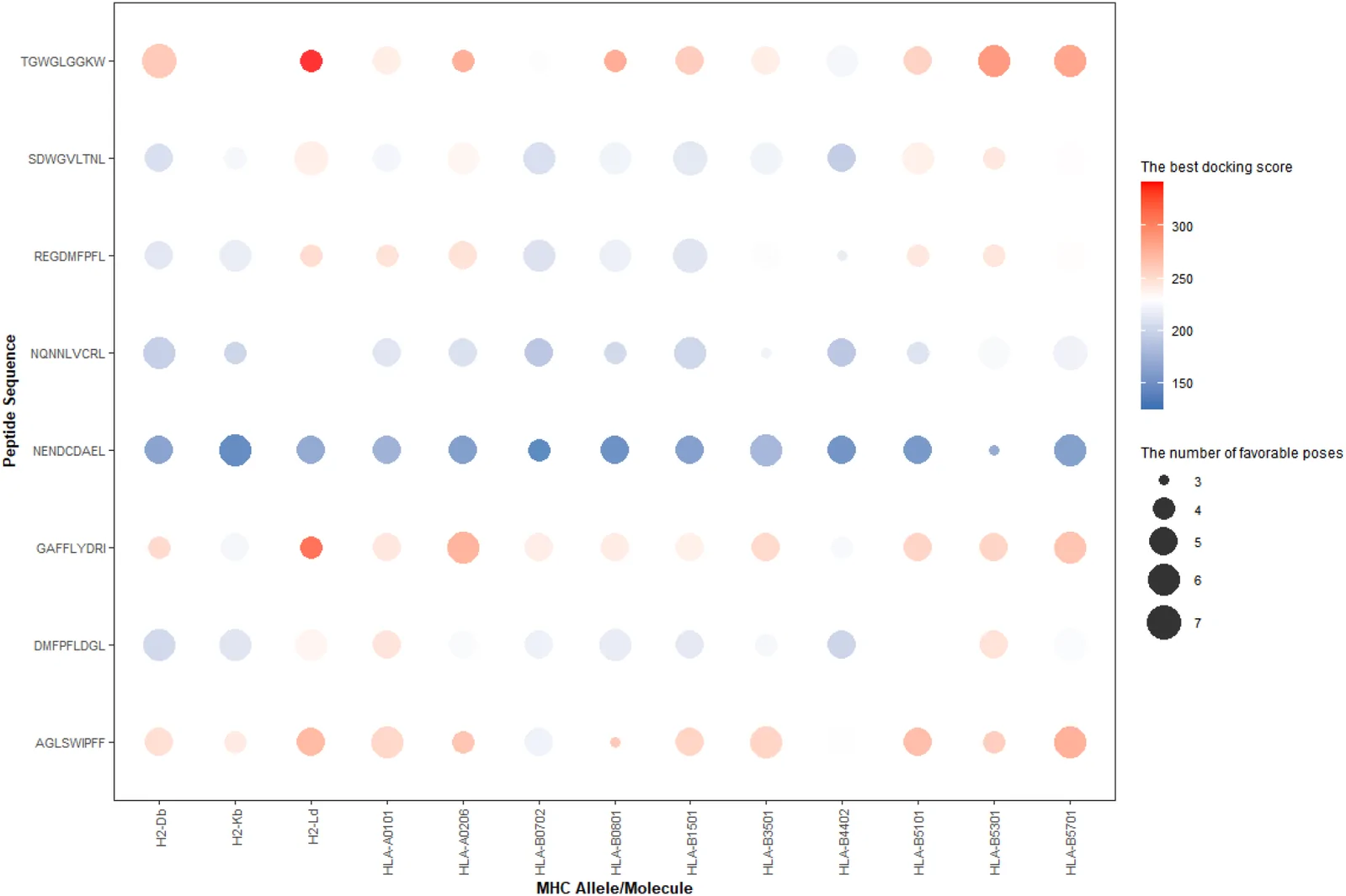
Bubble plot of epitope and different MHC-I docking scores. Red indicates more favorable (larger absolute-magnitude) HPEPDOCK scores, whereas blue indicates less favorable scores. Bubble size correlates with the number of HLA-I-bound epitope poses, with larger bubbles representing a greater number of favorable poses.

Based on the docking results, we selected 11 representative epitope-MHC complexes for comprehensive evaluation by integrating docking analyses with RMSD metrics from MD simulations. Detailed experimental results are summarized in **Supplementary Figures 2.1**–**2.13**. The relevant charts and representative structural snapshots fully reveal the dynamic behavioral characteristics of each complex. The short exploratory trajectories showed heterogeneous behavior across complexes. RMSD analysis revealed that, among the 11 epitope-MHC complexes evaluated, the combination of AGLSWIPFF with HLA-B*53:01 exhibited the lowest backbone RMSD, suggesting the highest conformational stability *in silico*. The molecular docking and MD results are presented only as descriptive structural observations and are not used as evidence of immunogenicity or biological efficacy.

### 3.7 Sequence alignment between Angola2005 and 94 other MARV strains

Analysis of 94 variant strains within the MARV species revealed six mutations located within the eight selected epitopes (**Figure 3A**). Three high-frequency mutation sites were identified among these mutations. The mutation probabilities were calculated: I149V (14.894%), R440K (13.830%), M444I (19.149%) (**Figure 3B**). For amino acid position 440, the R440K mutation was always accompanied by the M444I mutation.

**Figure 3:**
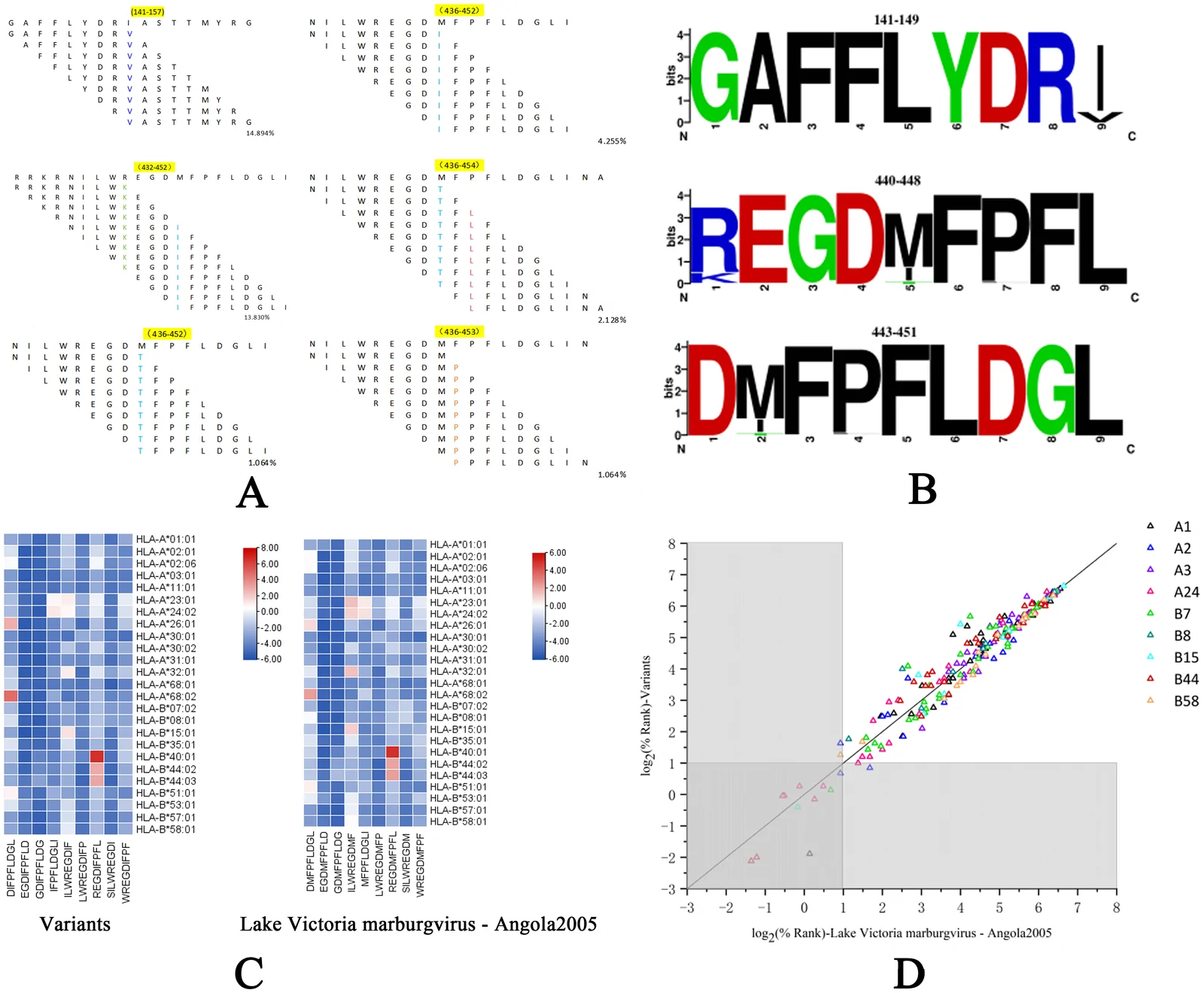
MARV high-affinity antigen fragment mutation comparison. **(A)** Distribution of all mutation sites and their corresponding frequencies. **(B)** WebLogo representation of three high-frequency mutation sites within the selected epitopes. **(C)** M444I: The heat map showing affinity changes before and after mutation at positions 436–452. Darker red colors indicated higher affinity, while lighter blue colors represented lower affinity. **(D)** M444I: Scatter plot comparing affinity before and after mutation at positions 436–452. The gray area denoted affinity rankings within the top 2%. Points closer to the diagonal line (Y=X) indicated greater similarity in affinity between the original and mutated sequences.

To investigate the effect of the mutation site on the affinity, we focused on the mutation site with the highest mutation frequency M444I (19.149%). A heat map and a scatter plot were generated to visualize affinity changes before and after mutation. In the heat map, warmer color indicated high-affinity (**Figure 3C**). In the scatter plot, the closer the scatter was to the diagonal line Y=X, the more similar the mutant was to the Lake Victoria marburgvirus Angola2005 affinity (**Figure 3D**). The heat maps and scatter plots of I149V and R440K are shown in **Supplementary Figure 3**.

### 3.8 Toxicity and allergenicity predictions indicate preliminary safety assessment of selected epitopes

The prediction results (**Table 7**) showed that one selected epitope (NENDCDAEL) was predicted to be allergenic. None of the selected epitopes were predicted to be toxic. Detailed results for all epitopes are presented in **Supplementary Tables 6**, **7**.

**Table 7:** Antigenic epitope allergenicity and toxicity prediction.

Protein	Sequence	Position	Allergenic	Toxic
GP1	GAFFLYDRI	141-149	N	N
GP2	REGDMFPFL	440-448	N	N
	DMFPFLDGL	443-451	N	N
	NENDCDAEL	508-516	A	N
	AGLSWIPFF	528-536	N	N
	NQNNLVCRL	551-559	N	N
	TGWGLGGKW	636-644	N	N
	SDWGVLTNL	647-655	N	N

### 3.9 ELISpot validation of selected epitopes

Responses to the epitopes REGDMFPFL, DMFPFLDGL, NENDCDAEL, AGLSWIPFF, TGWGLGGKW and SDWGVLTNL were strong, as indicated by high numbers of IFN-γ-producing cells. The epitopes GAFFLYDRI and NQNNLVCRL exhibited relatively strong responses. The results showed that the immune response of BALB/c mice was stronger than that of C57BL/6 mice. The epitopes DMFPFLDGL and NENDCDAEL elicited the highest IFN-γ secretion in both BALB/c and C57BL/6 mice, followed by TGWGLGGKW, AGLSWIPFF and SDWGVLTNL. BALB/c mice generally showed higher responses than C57BL/6 mice for several peptides (**Figure 4**). These findings were consistent with the *in silico* predictions, which indicated favorable binding of these epitopes to H-2d alleles. **Supplementary Figure 4** shows the negative control data of ELISpot and the comparison data with the experimental group, demonstrating that the epitopes can stimulate significant changes in immune responses in both BALB/c and C57BL/6 mice.

**Figure 4:**
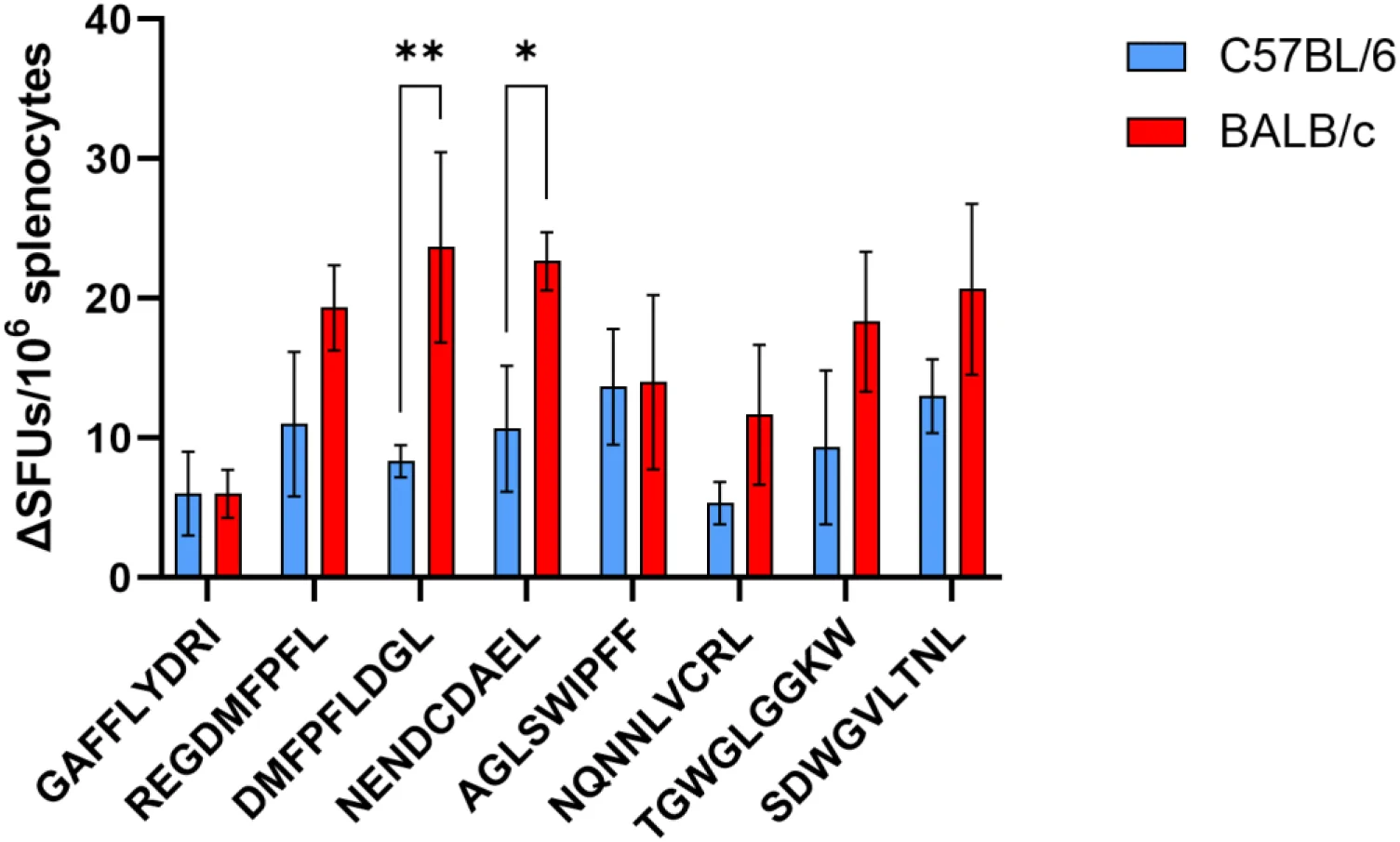
Histogram of epitope ELISpot result. Selected epitopes induced IFN-γ secretion from splenocytes in BALB/c and C57BL/6 mice. The experiment was performed in three independent batches, with two mice per strain per batch, yielding six biological replicates per strain. Each peptide and cell suspension was assayed in duplicate wells, and the mean of the duplicates was used for analysis. Two-way ANOVA revealed significant strain differences for DMFPFLDGL (**P = 0.0012) and NENDCDAEL (*P = 0.0158), with BALB/c responding more strongly than C57BL/6. *P < 0.05, **P < 0.01. The overall mean difference between the two strains was 7.375 (95% CI: 4.809 to 9.941).

### 3.10 ELISA validation of immune responses induced by selected epitopes

As shown in **Figure 5**, all eight selected peptides were recognized by IgG antibodies in the serum of immunized mice. Among all tested epitopes, DMFPFLDGL showed the highest absorbance values in both BALB/c and C57BL/6 mice. The remaining seven epitopes also exhibited robust immunostimulatory effects. These data demonstrate antibody recognition of the corresponding linear peptide sequences after full-length GP immunization. Similar to the ELISpot results, ELISA also showed slightly higher serum antibody responses in BALB/c mice than in C57BL/6 mice. The negative control data of ELISA and the comparison data with the experimental group are provided in **Supplementary Figure 4**.

**Figure 5:**
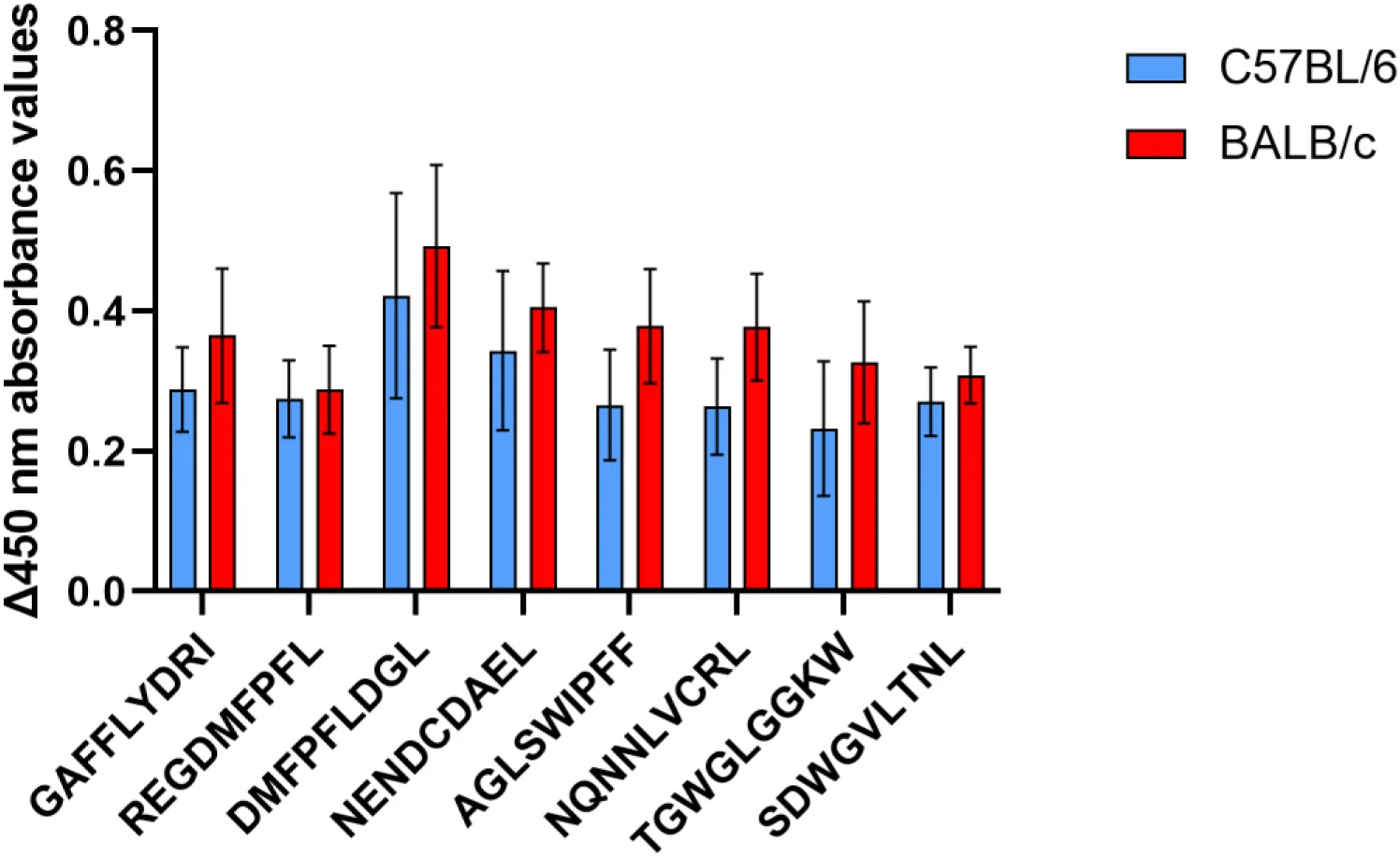
The selected epitopes in different mouse strains in the ELISA assay. ▲OD450 nm absorbance value was determined as the absorbance at 450 nm minus the negative control absorbance per well. The experiment was performed in three independent batches, with two mice per strain per batch, yielding six biological replicates per strain. Each epitope-serum combination was assayed in duplicate wells, and the mean of the duplicates was used for analysis. Two-way ANOVA revealed significant main effects of peptide (P<0.0001) and mouse strain (P<0.0001, 95% CI: −0.107 to −0.038), no significant interaction (P = 0.82), and no significant strain difference for any individual peptide (adjusted P>0.05 for all peptides).

### 3.11 Flow cytometry of cellular immune responses induced by selected epitopes in mice

To further investigate the cellular immune responses, flow cytometry was performed to assess cytokine secretion by CD8^+^ T cells in splenocytes isolated from BALB/c mice. The gating strategy is presented in **Figure 6A**, and IFN-γ and IL-2 responses are shown in **Figures 6B, C**. The results demonstrated that the levels of these cytokines were significantly higher in the experimental group compared to the control group (**Figures 6B, C**). Peptide-pool stimulation increased the frequencies of cytokine-positive CD8+ T cells relative to the matched control condition. These results support peptide-reactive CD8+ T-cell activation after GP immunization, further validating the functional capacity of the selected epitopes to induce specific cellular immunity *in vivo*.

**Figure 6:**
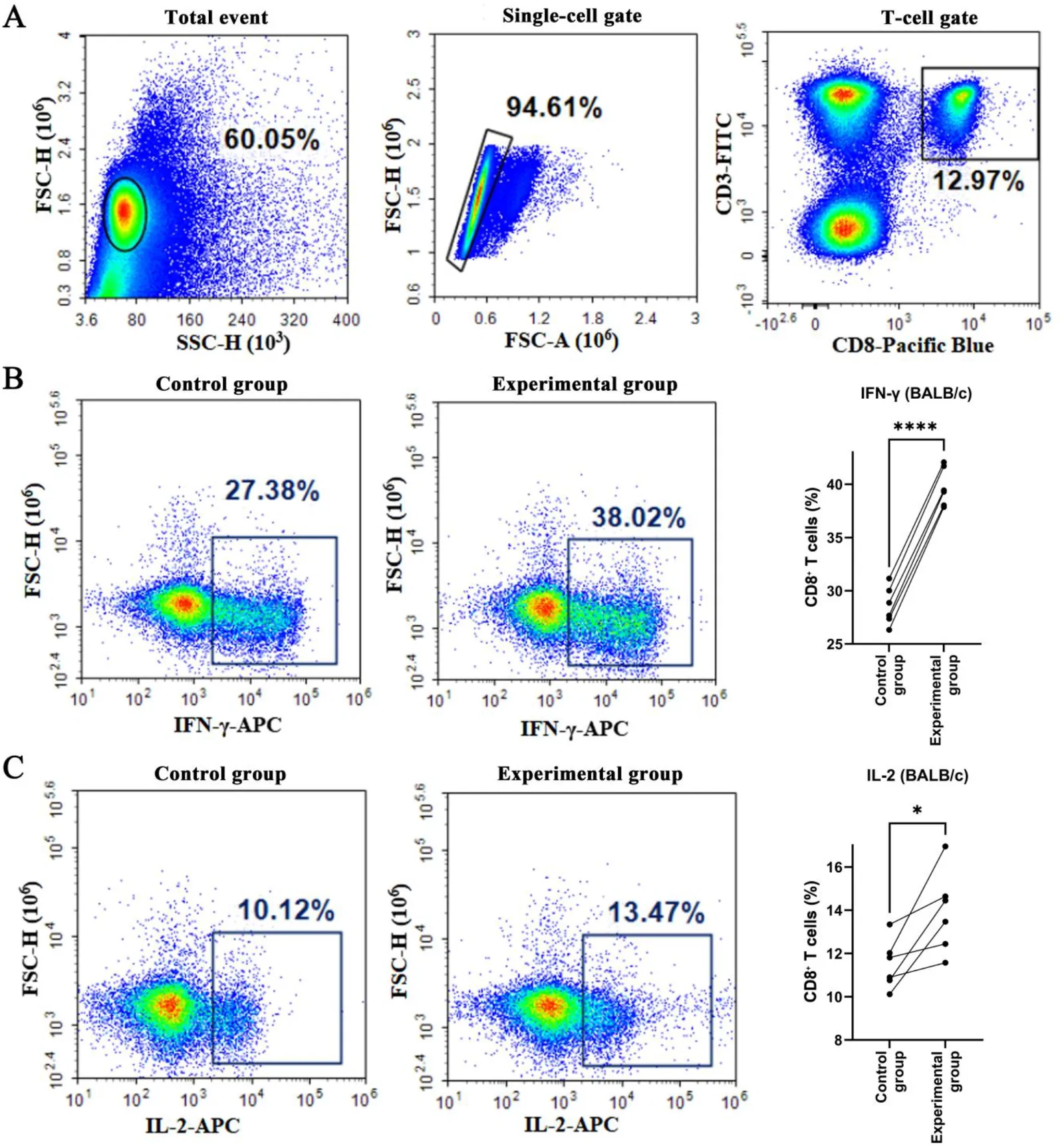
Cytokine expression by CD8^+^ T cells in BALB/c mice was evaluated using flow cytometry. **(A)** A gating strategy was established to identify cytokine expression in CD8^+^ T cells. **(B)** IFN-γ secretion in CD8^+^ T cells from BALB/c mice was significantly higher in the experimental group than in the control group (n=6 for control group, n=6 for experimental group). A two-tailed paired t test showed a significant increase in IFN-γ secretion in the experimental group (t = 53.26, df = 5, P < 0.0001, mean difference = 11.16%, 95% CI = 10.62–11.70%). **(C)** IL-2 secretion in CD8^+^ T cells from BALB/c mice was higher in the experimental group than in the control group (n=6 for control group, n=6 for experimental group). A two-tailed paired t test revealed a significant increase in IL-2 secretion in the experimental group (t = 3.295, df = 5, P = 0.0216, mean difference = 2.428, 95% CI = 0.534-4.323).

## 4 Discussion

MARV is one of the causative agents of lethal hemorrhagic fever and the GP is a critical target, as it effectively activates CD8^+^ and CD4^+^ T cells, eliciting specific immune responses (13). Furthermore, viral clearance is highly dependent on T cell immunity induced by vaccine, particularly IFN-γ and IL-2 secreted by CD8^+^ T cells (42). Therefore, we aimed to identify and validate potential immunodominant MHC-I-restricted T cell epitopes derived from the MARV GP. Our study advances immunoinformatic predictions by incorporating a combined computational and experimental approach for MARV GP epitope identification (19, 43, 44).

In this study, we employed a combined bioinformatics and immunological approach to screen for potentially MHC-I-restricted T cell epitopes derived from the MARV GP. We used five algorithms to predict MHC-I affinity, identifying 82 dominant epitopes specific to H-2 and 204 specific to HLA-I. The intersection of these two sets yielded 65 epitopes that exhibited affinity for both H-2 and HLA-I. Using two different computational tools, PAAQD 2.0 and IEDB, a total of 14 epitopes with high affinity for both murine and human MHC-I molecules and strong immunogenicity were further identified. A dominant epitope was considered conserved if it exhibited both intraspecific and intergeneric conservation. However, since the genus Orthomarburgvirus contained only one species, we defined conserved epitopes on the basis of intraspecific conservation only, rather than also requiring intergeneric conservation. To assess the conservation of the epitopes, we evaluated the screened epitopes. Ultimately, we obtained eight selected epitopes. These selected epitopes simultaneously exhibited high affinity for MHC-I molecules, strong immunogenicity, and high intraspecific conservation.

Molecular docking and MD simulations provided complementary structural characterization of the predicted peptide-MHC complexes. We prioritized molecular docking for high-throughput prediction of epitope-MHC-I binding conformations, as it offers an effective balance between computational efficiency and predictive performance. Based on the docking results, we selected complexes with favorable binding scores for further MD simulations to assess their dynamic stability. Notably, only a subset of these MD simulations yielded stable trajectories, indicating that docking alone cannot fully capture structural robustness. Bidirectional hierarchical clustering analysis revealed that certain members of the human HLA-I and H-2 share high similarity in presenting the same MARV GP epitopes. Sequence analysis across 94 MARV GP records revealed three high-frequency amino acid mutation sites within two of the eight selected epitopes. These mutations had a minimal impact on the affinity of the epitopes and the surrounding epitope sequences. We predicted the toxicity and allergenicity of the eight selected epitopes, identifying one as potentially allergenic and none as potentially toxic. The epitope NENDCDAEL was predicted to be allergenic, which poses a safety risk in clinical translation. This risk could be addressed by excluding NENDCDAEL from multivalent vaccine formulations or by exploring sequence and formulation modifications to reduce its allergenic potential. Any modification would require reassessment of MHC-I binding and immunogenicity. Importantly, the predicted allergenicity is based solely on computational analysis and should be further evaluated in appropriate preclinical allergy models before clinical development (45).

The eight epitopes were selected as stimuli for ELISpot assays to verify their capacity to induce IFN-γ secretion by CD8^+^ T cells. DMFPFLDGL and NENDCDAEL elicited the strongest responses. ELISA experiments further suggested that immunization with the full-length MARV GP generated antibodies which recognized several of the selected linear peptide sequences. Among them, DMFPFLDGL elicited the highest antibody response, as reflected by the highest absorbance values in the ELISA assays. Flow cytometry with the pooled peptides showed increased IFN-γ and IL-2 production in CD8+ T cells. In summary, through a combination of *in silico* predictions and experimental validation, we identified DMFPFLDGL (aa 443-451) as the epitope that elicited the strongest serum immune responses among the eight selected epitopes. Although the selected peptides were identified as MHC-I-restricted T-cell epitope candidates, several were also recognized by IgG in sera from mice immunized with full-length MARV GP, particularly DMFPFLDGL. Importantly, the peptides themselves were not used for immunization. Therefore, the ELISA results do not demonstrate that these 9-mer peptides independently induce B-cell activation or humoral immunity. Rather, they indicate that GP-induced antibodies can recognize the corresponding linear peptide sequences. Thus, the ELISA findings are considered complementary to, rather than mechanistic evidence for, the primary MHC-I-restricted CD8^+^ T-cell responses investigated in this study.

The eight MHC-I-restricted epitopes identified in this study effectively activate CD8^+^ T cells. Compared with the previous study, one of the MHC-I-restricted epitopes screened in this study was completely identical to the known epitope REGDMFPFL (aa 440–448) derived from the GP of the Angola strain. This finding partially validates the reliability of our prediction strategy (19). Additionally, the epitope we identified SDWGVLTNL (aa 647–655) partially overlaps with the previously reported epitope LTNLGILLL (aa 652–660), further suggesting the presence of a conserved immunogenic region within this GP segment (46). Our finding also provides complementary insights into the recently reported MARV-neutralizing antibody MARV16. MARV16 achieves potent viral neutralization by recognizing a prefusion-specific epitope spanning GP1 and GP2, but clearance of infected cells still relies on T cell immunity (47). Similarly, a recent study identified the conserved GP1–GP2 wing region as a neutralizing immunogen in MARV survivors, reinforcing the vaccine potential of this interface (48). Therefore, combining T cell epitope-based vaccines with neutralizing antibodies may provide a more comprehensive strategy against MARV infection. Moreover, a recent study demonstrated that the GP2 wing region is subject to glycan shielding that affects antibody recognition (49). However, we acknowledge that glycosylation may also affect T cell epitope processing. Although our epitopes lack N-X-S/T motifs, we did not experimentally exclude O-glycan interference and other types of glycosylation (50).

Nevertheless, this study has certain limitations. First, at the computational level, our screening focused on epitope–MHC-I binding affinity and structural stability. However, we did not incorporate upstream antigen-processing steps such as proteasomal cleavage and TAP transport, which are critical for *in vivo* epitope generation (51). Moreover, molecular docking and MD simulations provided useful structural information regarding binding stability, but do not directly predict immunogenicity or biological efficacy. Second, at the animal model level, three predicted epitopes in Section 3.3 showed favorable HLA-I binding, immunogenicity and sequence conservation, but were not retained for mouse validation because they lacked H-2 binding. Future studies could employ humanized HLA-I transgenic mouse models or *in vitro* T cell activation assays to evaluate their immunogenic properties. Although the mice were housed under specific-pathogen-free conditions, elevated background responses were observed in some ELISpot negative-control wells, possibly reflecting non-specific baseline immune activation. This background represents a potential confounding factor and may reduce the sensitivity and precision of peptide-specific response estimates, particularly for weakly responding epitopes. Accordingly, the weak responses should be interpreted cautiously. Nevertheless, responses that were consistently distinguishable from their corresponding controls remain valid. Third, at the population coverage level, the extensive polymorphism of HLA-I molecules was not fully represented. The combined coverage of the eight epitopes was below 70% in South America (63.19%), South Asia (62.91%), and Northeast Asia (64.08%), and was 73.58% in Africa. These gaps could be addressed by incorporating additional epitopes from other MARV proteins or by screening other HLA-I-restricted epitopes in future studies. Finally, at the translational potential level, the assays used in this study evaluate immunogenicity but do not establish protective efficacy. No MARV challenge experiments were performed. Therefore, whether these epitopes confer protection against infection remains unknown.

Our findings suggest that these epitopes may hold potential as components of future MARV vaccines. The epitopes are applicable to multiple vaccine platforms. For epitope-based vaccines, they can be directly synthesized and coupled with an adjuvant. For DNA-based vaccines, the current full-length GP expression cassette can be further optimized by enhancing the expression of these eight epitopes, which may strengthen CD8^+^ T-cell responses without requiring the entire GP. For multi-epitope vaccines, inclusion of additional B-cell and CD4^+^ T-cell epitopes would broaden immune coverage (46, 52). Future development should determine antigen processing and HLA-restricted presentation, confirm immunogenicity in human-relevant systems, optimize epitope combinations and delivery platforms, and directly test protection in appropriate challenge models.

## 5 Conclusion

In summary, our study identified 8 MARV GP-derived epitopes with high affinity for HLA-I and H-2 alleles, strong immunogenicity and high conservation through integrated bioinformatics analysis of multiple databases. These GP-derived epitopes may serve as candidates for multivalent vaccine design and further investigation of the T cell immune mechanisms against MARV. Subsequent work will focus on extending validation to humanized HLA-I transgenic mouse models, and exploring the integration of these epitopes with suitable adjuvants to create optimized multivalent immunogens. Their protective efficacy against MARV will be systematically evaluated.

## Data Availability

The original contributions presented in the study are included in the article/**Supplementary Material**, further inquiries can be directed to the corresponding authors.
