# Supplementary material for: Integrated immunoreactivity analysis of MHC-I epitopes derived from the Marburg virus glycoprotein
Source: Front Immunol. 2026 Oct 6;17:1902130. doi: 10.3389/fimmu.2026.1902130 (PMC13680160; doi:10.3389/fimmu.2026.1902130)
Supplement: Supplementary file 1 [file DataSheet1.docx]

Supplementary Material

# 1 Supplementary Figure

**Supplementary Figure 1. Bidirectional hierarchical clustering of predicted MARV GP 9-mer peptide binding profiles across MHC-I molecules.**

Thirty-one MHC-I molecules were assigned to three clusters, namely an HLA-I exclusive cluster, an H2 exclusive cluster and a cross-reactive cluster (HLA major). The heat map summarizes similarities and differences in predicted peptide-binding profiles across MHC-I molecules. Red indicates relatively stronger predicted affinity and blue indicates relatively weaker predicted affinity within the displayed scale.

**Supplementary Figure 2.1**. Comparison of epitope-MHC RMSD distributions among simulated pMHC complexes. Box plots show the distributions of epitope Root Mean Square Deviation (RMSD, Å) for each pair, ordered by their median RMSD values to illustrate the spectrum of conformational dynamics within the binding groove. The dashed lines indicate the thresholds for very stable (< 2 Å, red) and stable (< 5 Å, orange) complexes. The results highlight the exceptional structural rigidity of the AGLSWIPFF peptide when bound to HLA-B*53:01 and HLA-B*51:01, with RMSD values remaining consistently below the 2 Å threshold. In contrast, several complexes exhibit high RMSD values exceeding 25 Å, indicating substantial deviation from the initial conformation.

**Supplementary Figure 2.2**. Mean peptide RMSD across simulated pMHC complexes. Bars represent the mean steady-state peptide RMSD (Å) for each combination, with error bars indicating standard deviation. Complexes are color-coded according to their conformational stability: steelblue for highly stable, purple for moderately stable, and salmon for flexible.

MD Analysis of HLA-B*53:01 AGLSWIPFF complex

A	B

C	D

**Supplementary Figure 2.3.** MD Analysis of HLA-B*53:01 AGLSWIPFF complex

(A) Backbone RMSD: the complex backbone RMSD trace experienced fluctuations and significant jumps before 1.3 ns, then stabilized at a high level around 10 Å, reflecting a major conformational transition.

(B) Backbone RMSF: Most regions showed relatively low fluctuations, whereas the terminal region (after approximately atom index 3100) exhibited substantially greater flexibility, with values exceeding 25 Å.

(C) PCA projection: PCA of backbone motions showed two clearly separated conformational clusters, indicating sampling of distinct conformational states during the simulation.

(D) Radius of gyration: after large oscillations in the early stage, the radius of gyration dropped sharply at 1.3 ns and then stabilized. This suggested that the complex ultimately became more compact than in its initial state.

MD Analysis of HLA-B*51:01 AGLSWIPFF complex

A	B

C	D

**Supplementary Figure 2.4.** MD Analysis of HLA-B*51:01 AGLSWIPFF complex

(A) Backbone RMSD: the trajectory reached an approximately 2.2 Å plateau after about 1 ns, indicating relatively limited subsequent deviation from the aligned reference structure.

(B) Backbone RMSF: most positions showed low fluctuations (generally below approximately 1.5 Å), with greater motion confined to localized regions.

(C) PCA projection: frames converged toward a relatively well-defined conformational cluster over the analyzed trajectory, consistent with restricted conformational sampling.

(D) Radius of gyration: the complex maintained a relatively stable radius of gyration of approximately 24.1 Å during the analyzed interval.

MD Analysis of H2-L^d^ GAFFLYDRI complex

A	B

C	D

**Supplementary Figure 2.5.** MD Analysis of H2-L^d^ GAFFLYDRI complex

(A) Backbone RMSD: the system remained below 5 Å before 4.8 ns, then jumped to over 40 Å, suggesting a large conformational deviation relative to the initial structure.
(B) Backbone RMSF: the complex backbone RMSF along the sequence exhibited significantly high fluctuations (peaks up to 15 Å) in the region of atom indices 3000 to 6000, indicating that this part of the structure is highly flexible.
(C) PCA projection: PCA of backbone motions showed that the sampled points formed a distinct, isolated cluster, indicating that the complex underwent a large-scale conformational transition.
(D) Radius of gyration: marked oscillations occurred near the end of the trajectory, indicating a loss of structural compactness in the analyzed simulation.

MD Analysis of H2-L^d^ TGWGLGGKW complex

A	B

C	D

**Supplementary Figure 2.6.** MD Analysis of H2-L^d^ TGWGLGGKW complex

(A) Backbone RMSD: fluctuations between 8 Å and 26 Å reflected a highly flexible trajectory.

(B) Backbone RMSF: localized regions of flexibility with fluctuations exceeding 40 Å suggested substantial conformational fluctuations.

(C) PCA projection: multiple widely isolated clusters were observed, indicating substantial conformational heterogeneity in the sampled trajectory.

(D) Radius of gyration: abrupt changes between 54 Å and 59 Å were consistent with a non-compact and fluctuating structural state throughout the simulation.

MD Analysis of HLA-B*53:01 TGWGLGGKW complex

A	B

C	D

**Supplementary Figure 2.7.** MD Analysis of HLA-B*53:01 TGWGLGGKW complex

(A) Backbone RMSD: the trace showed frequent jumps between approximately 2 Å and 10 Å, reflecting significant conformational switching or trajectory discontinuity during the simulation.

(B) Backbone RMSF: most regions of the protein maintained low flexibility, with fluctuations observed only in the terminal regions, indicating that the core structure is relatively stable.

(C) PCA projection: Two distinct clusters in phase space indicated discontinuous conformational sampling.

(D) Radius of gyration: stepwise changes in the radius of gyration between 23.2 Å and 24.5 Å reflected transitions of the complex between states of different compactness.

MD Analysis of HLA-B*53:01 DMFPFLDGL complex

A	B

C	D

**Supplementary Figure 2.8.** MD Analysis of HLA-B*53:01 DMFPFLDGL complex

(A) Backbone RMSD: the system entered a relatively high plateau after initial fluctuations, reflecting significant conformational switching followed by subsequent stabilization during the simulation.

(B) Backbone RMSF: the core regions of the protein maintained low flexibility, with fluctuations observed only in the terminal regions.

(C) PCA projection: PCA of backbone motions showed that the sampled points formed two clearly separated clusters in phase space, indicating that the complex transitions between two major conformational states.

(D) Radius of gyration: following an initial adjustment period, the radius of gyration became relatively stable, suggesting maintenance of a compact conformation thereafter.

MD Analysis of HLA-B*35:01 AGLSWIPFF complex

A	B

C	D

**Supplementary Figure 2.9.** MD Analysis of HLA-B*35:01 AGLSWIPFF complex

(A) Backbone RMSD: frequent jumps between multiple discrete plateaus were observed, reflecting substantial conformational fluctuations and discontinuous changes during the simulation.

(B) Backbone RMSF: relatively high fluctuations were observed across broad portions of the analyzed structure.

(C) PCA projection: multiple highly separated and discrete clusters in phase space indicated that the complex underwent large-scale conformational transitions.

(D) Radius of gyration: the radius of gyration oscillated dramatically between 23 Å and 45 Å, suggesting that the complex underwent significant expansion and structural loosening during the simulation.

MD Analysis of HLA-A*02:06 GAFFLYDRI complex

A	B

C	D

**Supplementary Figure 2.10.** MD Analysis of HLA-A*02:06 GAFFLYDRI complex

(A) Backbone RMSD: frequent changes between 3 Å and 26 Å reflected conformational instability during the simulation from 0 ns to 3 ns.

(B) Backbone RMSF: multiple regions showed high fluctuation values (peaks up to 25 Å), indicating significant overall flexibility of the protein.

(C) PCA projection: two widely separated and completely distinct clusters suggested that the complex underwent large-scale transitions between distinctly different conformational states.

(D) Radius of gyration: rapid switching between 25 Å and 36 Å demonstrated that the HLA-A*02:06 GAFFLYDRI complex experienced dramatic structural expansion and contraction during the simulation.

MD Analysis of HLA-A*02:06 TGWGLGGKW complex

A	B

C	D

**Supplementary Figure 2.11.** MD Analysis of HLA-A*02:06 TGWGLGGKW complex

(A) Backbone RMSD: a jump from 2 Å to over 27 Å at approximately 0.7 ns reflected discontinuous and large conformational changes during the simulation.

(B) Backbone RMSF: high fluctuations (peaks near 35 Å) across the entire sequence indicated increased conformational flexibility during the simulation.

(C) PCA projection: three widely separated and completely distinct clusters in phase space indicated that the system underwent abrupt conformational switching during the simulation.

(D) Radius of gyration: stepwise changes between 23 Å and 36 Å reflected structural expansion and contraction of the HLA-A*02:06 TGWGLGGKW complex during the simulation.

MD Analysis of HLA-A*01:01 AGLSWIPFF complex

A	B

C	D

**Supplementary Figure 2.12.** MD Analysis of HLA-A*01:01 AGLSWIPFF complex

(A) Backbone RMSD: a dramatic jump at 1 ns followed by a sustained high value of approximately 28 Å reflected significant conformational transitions or instability during the simulation.
(B) Backbone RMSF: high fluctuations (peaks approaching 40 Å) in the middle region of the sequence indicated strong flexibility in specific structural domains of the protein.
(C) PCA projection: multiple highly separated and discrete clusters indicated that the complex underwent discontinuous large-scale conformational switching during the simulation.
(D) Radius of gyration: it stabilized after initial oscillations, indicating that the HLA-A*01:01 AGLSWIPFF complex experienced notable conformational changes during the simulation.

MD Analysis of H2-D^b^ TGWGLGGKW complex

A	B

C	D

**Supplementary Figure 2.13.** MD Analysis of H2-D^b^ TGWGLGGKW complex

(A) Backbone RMSD: a jump from 2 Å to over 28 Å accompanied by transient peaks at the beginning of the simulation reflected significant conformational discontinuity and instability.
(B) Backbone RMSF: high atomic fluctuations (peaks exceeding 16 Å) across the entire sequence indicated that the overall structure of the protein was highly flexible.
(C) PCA projection: four highly dispersed and completely separated clusters in phase space indicated that the complex underwent abrupt large-scale conformational transitions.

(D) Radius of gyration: frequent fluctuations between 26 Å and 39 Å reflected instability in the structural compactness of the H2-D^b^ TGWGLGGKW complex during the simulation.

**Supplementary Figure 3. Predicted binding-affinity changes for the I149V and R440K mutations.**

The heat maps and scatter plots compare predicted binding-affinity patterns before and after the I149V and R440K substitutions. The observed changes were limited in the analyzed prediction framework.

**Supplementary Figure 4. ELISpot and ELISA negative control results and the comparisons with the experimental group.**

**(A)** ELISA negative control and experimental comparisons: Serum antibody levels were evaluated by measuring absorbance values at 450 nm against the indicated peptides. In BALB/c mice, one-way ANOVA revealed significant overall differences (P < 0.0001). Šídák's multiple comparisons test showed that all eight peptides significantly higher absorbance values compared with the negative control (P < 0.0001 for each). In C57BL/6 mice, ANOVA also showed significance (P < 0.0001), and all peptides yielded P < 0.0001 compared with the negative control.

**(B)** ELISpot negative control and experimental comparisons: IFN-γ spot-forming cells were quantified per 1 × 10^6^ splenocytes following peptide stimulation. In C57BL/6 mice, one-way ANOVA showed a significant overall difference among the groups (P = 0.0247). Šídák's multiple comparisons test showed that the following peptides elicited significantly stronger IFN-γ responses than the negative control: REGDMFPFL (P = 0.0456), NENDCDAEL (P = 0.0369), AGLSWIPFF (P = 0.0082), SDWGVLTNL (P = 0.0126). In BALB/c mice, one-way ANOVA showed a significant overall difference among the groups (P = 0.0014). Šídák's multiple comparisons test showed that the following peptides elicited significantly stronger IFN-γ responses than the negative control: REGDMFPFL (P = 0.0291), DMFPFLDGL (P = 0.0014), NENDCDAEL (P = 0.0022), TGWGLGGKW (P = 0.0129), SDWGVLTNL (P = 0.0049).

Statistical significance was determined by comparison with the negative control group (*p < 0.05, **p < 0.01, ***p < 0.001, ****p < 0.0001).
