# Supplementary material for: Integrated immunoreactivity analysis of MHC-I epitopes derived from the Marburg virus glycoprotein
Source: Front Immunol. 2026 Oct 6;17:1902130. doi: 10.3389/fimmu.2026.1902130 (PMC13680160; doi:10.3389/fimmu.2026.1902130)
Supplement: Supplementary file 2 [file DataSheet2.docx]

Supplementary Material

# 1 Supplementary Tables

Supplementary Table 1. List of 94 glycoprotein (GP) accession numbers of Marburg strains used in analysis.

| **Accession Number** | **Protein Type** | **Strain Name** |
| --- | --- | --- |
| AAC4458.1 | glycoprotein | glycoprotein precursor Orthomarburgvirus marburgense |
| AAC4459.1 | glycoprotein | glycoprotein precursor Orthomarburgvirus marburgense |
| AAC446.1 | glycoprotein | glycoprotein precursor Orthomarburgvirus marburgense |
| AAQ55258.1 | glycoprotein | GP protein Orthomarburgvirus marburgense |
| AAR85456.1 | glycoprotein | GP protein Orthomarburgvirus marburgense |
| AAR85463.1 | glycoprotein | GP protein Orthomarburgvirus marburgense |
| ABA87127.2 | glycoprotein | glycoprotein Marburg virus - Musoke Kenya 198 |
| ABS17551.1 | glycoprotein | glycoprotein Ravn virus - Ravn Kenya 1987 |
| ABS17558.1 | glycoprotein | glycoprotein Lake Victoria marburgvirus - Ci67 |
| ACD135.1 | glycoprotein | glycoprotein Ravn virus - Ravn Kenya 1987 |
| ACD1312.1 | glycoprotein | glycoprotein Ravn virus - Ravn Kenya 1987 |
| ACT7921.1 | glycoprotein | glycoprotein Orthomarburgvirus marburgense |
| ACT7928.1 | glycoprotein | glycoprotein Orthomarburgvirus marburgense |
| ACT79215.1 | glycoprotein | glycoprotein Orthomarburgvirus marburgense |
| ACT79222.1 | glycoprotein | glycoprotein Orthomarburgvirus marburgense |
| ACT79229.1 | glycoprotein | glycoprotein Orthomarburgvirus marburgense |
| ACT79236.1 | glycoprotein | glycoprotein Orthomarburgvirus marburgense |
| ACT79243.1 | glycoprotein | glycoprotein Orthomarburgvirus marburgense |
| ADM72984.1 | glycoprotein | GP Orthomarburgvirus marburgense |
| ADM72991.1 | glycoprotein | GP Orthomarburgvirus marburgense |
| ADM72998.1 | glycoprotein | GP Orthomarburgvirus marburgense |
| AEW11937.1 | glycoprotein | glycoprotein Lake Victoria marburgvirus - Leiden |
| AFV3114.1 | glycoprotein | glycoprotein Orthomarburgvirus marburgense |
| AFV31146.1 | glycoprotein | glycoprotein Orthomarburgvirus marburgense |
| AFV31153.1 | glycoprotein | glycoprotein Orthomarburgvirus marburgense |
| AFV3116.1 | glycoprotein | glycoprotein Orthomarburgvirus marburgense |
| AFV31167.1 | glycoprotein | glycoprotein Orthomarburgvirus marburgense |
| AFV31174.1 | glycoprotein | glycoprotein Orthomarburgvirus marburgense |
| AFV31181.1 | glycoprotein | glycoprotein Orthomarburgvirus marburgense |
| AFV31188.1 | glycoprotein | glycoprotein Orthomarburgvirus marburgense |
| AFV31195.1 | glycoprotein | glycoprotein Orthomarburgvirus marburgense |
| AFV3122.1 | glycoprotein | glycoprotein Orthomarburgvirus marburgense |
| AFV3129.1 | glycoprotein | glycoprotein Orthomarburgvirus marburgense |
| AFV31216.1 | glycoprotein | glycoprotein Orthomarburgvirus marburgense |
| AFV31223.1 | glycoprotein | glycoprotein Orthomarburgvirus marburgense |
| AFV3123.1 | glycoprotein | glycoprotein Orthomarburgvirus marburgense |
| AFV31237.1 | glycoprotein | glycoprotein Orthomarburgvirus marburgense |
| AFV31244.1 | glycoprotein | glycoprotein Orthomarburgvirus marburgense |
| AFV31251.1 | glycoprotein | glycoprotein Orthomarburgvirus marburgense |
| AFV31258.1 | glycoprotein | glycoprotein Orthomarburgvirus marburgense |
| AFV31265.1 | glycoprotein | glycoprotein Orthomarburgvirus marburgense |
| AFV31272.1 | glycoprotein | glycoprotein Orthomarburgvirus marburgense |
| AFV31279.1 | glycoprotein | glycoprotein Orthomarburgvirus marburgense |
| AFV31286.1 | glycoprotein | glycoprotein Orthomarburgvirus marburgense |
| AFV31293.1 | glycoprotein | glycoprotein Orthomarburgvirus marburgense |
| AFV313.1 | glycoprotein | glycoprotein Orthomarburgvirus marburgense |
| AFV3137.1 | glycoprotein | glycoprotein Orthomarburgvirus marburgense |
| AFV31314.1 | glycoprotein | glycoprotein Orthomarburgvirus marburgense |
| AFV31321.1 | glycoprotein | glycoprotein Orthomarburgvirus marburgense |
| AFV31328.1 | glycoprotein | glycoprotein Orthomarburgvirus marburgense |
| AFV31335.1 | glycoprotein | glycoprotein Orthomarburgvirus marburgense |
| AFV31342.1 | glycoprotein | glycoprotein Orthomarburgvirus marburgense |
| AFV31349.1 | glycoprotein | glycoprotein Orthomarburgvirus marburgense |
| AFV31356.1 | glycoprotein | glycoprotein Orthomarburgvirus marburgense |
| AFV31363.1 | glycoprotein | glycoprotein Orthomarburgvirus marburgense |
| AFV3137.1 | glycoprotein | glycoprotein Orthomarburgvirus marburgense |
| AIL25245.1 | glycoprotein | glycoprotein Orthomarburgvirus marburgense |
| AJD39271.1 | glycoprotein | GP Orthomarburgvirus marburgense |
| AJD39278.1 | glycoprotein | GP Orthomarburgvirus marburgense |
| AJD39285.1 | glycoprotein | GP Orthomarburgvirus marburgense |
| AJY53714.1 | glycoprotein | virion spike glycoprotein precursor Orthomarburgvirus marburgense |
| AKB9572.1 | glycoprotein | envelope glycoprotein Orthomarburgvirus marburgense |
| AKI84283.1 | glycoprotein | envelope glycoprotein Orthomarburgvirus marburgense |
| ALS54754.1 | glycoprotein | envelope glycoprotein Orthomarburgvirus marburgense |
| AMZ481.1 | glycoprotein | virion spike glycoprotein Orthomarburgvirus marburgense |
| AMZ488.1 | glycoprotein | virion spike glycoprotein Orthomarburgvirus marburgense |
| APQ46224.1 | glycoprotein | glycoprotein Orthomarburgvirus marburgense |
| APQ46232.1 | glycoprotein | glycoprotein Orthomarburgvirus marburgense |
| APT69537.1 | glycoprotein | envelope glycoprotein Orthomarburgvirus marburgense |
| AWO14197.1 | glycoprotein | surface glycoprotein Orthomarburgvirus marburgense |
| CAA4857.1 | glycoprotein | structural protein Orthomarburgvirus marburgense |
| CAA78117.1 | glycoprotein | glycoprotein Marburg virus - Musoke Kenya 198 |
| CAA82539.1 | glycoprotein | GP protein Orthomarburgvirus marburgense |
| prf\|\|1914146 | glycoprotein | A GP protein |
| QBB2442.1 | glycoprotein | virion spike glycoprotein precursor Orthomarburgvirus marburgense |
| QBB2449.1 | glycoprotein | virion spike glycoprotein precursor Orthomarburgvirus marburgense |
| QDH1285.1 | glycoprotein | GP Orthomarburgvirus marburgense |
| QGX74971.1 | glycoprotein | glycoprotein Orthomarburgvirus marburgense |
| QGX74978.1 | glycoprotein | glycoprotein Orthomarburgvirus marburgense |
| QGX74985.1 | glycoprotein | glycoprotein Orthomarburgvirus marburgense |
| QGX74992.1 | glycoprotein | glycoprotein Orthomarburgvirus marburgense |
| QIC36348.1 | glycoprotein | glycoprotein Orthomarburgvirus marburgense |
| QIC36355.1 | glycoprotein | glycoprotein Orthomarburgvirus marburgense |
| QPJ5864.1 | glycoprotein | GP Orthomarburgvirus marburgense |
| sp\|P35253.1 | glycoprotein | \|VGP MABVM RecName: Full=Envelope glycoprotein AltName: Full=GP1 2 Short=GP AltName: Full=Virion spike glycoprotein Contains: RecName: Full=GP1 Contains: RecName: Full=GP2 Flags: Precursor |
| sp\|P35254.1 | glycoprotein | \|VGP MABVP RecName: Full=Envelope glycoprotein AltName: Full=GP1 2 Short=GP AltName: Full=Virion spike glycoprotein Contains: RecName: Full=GP1 Contains: RecName: Full=GP2 Flags: Precursor |
| sp\|Q1 | glycoprotein | PDC7.1\|VGP MABVR RecName: Full=Envelope glycoprotein AltName: Full=GP1 2 Short=GP AltName: Full=Virion spike glycoprotein Contains: RecName: Full=GP1 Contains: RecName: Full=GP2 Flags: Precursor |
| sp\|Q6 | glycoprotein | UY66.1\|VGP MABVO RecName: Full=Envelope glycoprotein AltName: Full=GP1 2 Short=GP AltName: Full=Virion spike glycoprotein Contains: RecName: Full=GP1 Contains: RecName: Full=GP2 Flags: Precursor |
| ULP54988.1 | glycoprotein | glycoprotein Orthomarburgvirus marburgense |
| WJN65217.1 | glycoprotein | surface glycoprotein Orthomarburgvirus marburgense |
| WKR37925.1 | glycoprotein | glycoprotein Orthomarburgvirus marburgense |
| WKR37932.1 | glycoprotein | glycoprotein Orthomarburgvirus marburgense |
| YP 1531156.1 | glycoprotein | glycoprotein Orthomarburgvirus marburgense |
| YP 955225.1 | glycoprotein | glycoprotein Orthomarburgvirus marburgense |

Supplementary Table 2. Comprehensive database versions and web servers.

| **Method** | **Database Version** | **Web Server** |
| --- | --- | --- |
| Affinity analysis | IEDB | https://www.iedb.org/ |
|  | NetMHCpan4. 1 | https://services.healthtech.dtu.dk/services/NetMHCpan-4. 1/ |
|  | SMMPMBEC | http://tools.immuneepitope.org/mhci/ |
|  | SYFPEITHI | http://www.syfpeithi.de/bin/MHCServer.dll/EpitopePrediction.htm |
|  | Rankpep | http://imed.med.ucm.es/Tools/rankpep.html |
| Immunogenicity analysis | IEDB | <http://tools.iedb.org/immunogenicity/> |
|  | PAAQD 2.0 | http://pirun.ku.ac.th/~fsciiok/PAAQD.rar |
| Conservation analysis | BLASTP | <https://blast.ncbi.nlm.nih.gov/Blast.cgi> |
| Molecular docking | HPEPDOCK 2.0 | <http://huanglab.phys.hust.edu.cn/hpepdock/> |
|  | RCSB PDB | <https://www.rcsb.org/> |
|  | NAMD 3.0 | https://www.ks.uiuc.edu/Development/Download/download.cgi?PackageName=NAMD |
|  | VMD | https://www.ks.uiuc.edu/Development/Download/download.cgi?PackageName=VMD |
| Population coverage analysis | IEDB | <http://tools.iedb.org/population/> |
| Sequence alignment of Marburg variants | WebLogo | <http://weblogo.berkeley.edu/logo.cgi> |
| Toxicity and allergenicity analysis | ToxinPred3.0 | <https://webs.iiitd.edu.in/raghava/toxinpred3/prediction.php> |
|  | AlgPred 2.0 | <https://webs.iiitd.edu.in/raghava/algpred2/> |

Supplementary Table 3. MHC-I allele included in the affinity analysis.

| **MHC-I** | **Supertype** | **Subtype** |
| --- | --- | --- |
| HLA-I | HLA-A1 | HLA-A*01:01、A*26:01、A*30:01、A*30:02 |
|  | HLA-A2 | HLA-A*02:01、A*02:03、A*02:06、A*68:02 |
|  | HLA-A3 | HLA-A*03:01、A*11:01、A*30:01、A*31:01、A*33:01、A*68:01 |
|  | HLA-A24 | HLA-A*23:01、A*24:02、A*32:01 |
|  | HLA-B7 | HLA-B*07:02、B*35:01、B*51:01、B*53:01 |
|  | HLA-B8 | HLA-B*08:01 |
|  | HLA-B15 | HLA-B*15:01 |
|  | HLA-B44 | HLA-B*40:01、B*44:02、B*44:03 |
|  | HLA-B58 | HLA-B*57:01、B*58:01 |
| H2 | H2-D^b^ | |
|  | H2-D^d^ | |
|  | H2-K^b^ | |
|  | H2-K^d^ | |
|  | H2-K^k^ | |
|  | H2-L^d^ | |

Supplementary Table 4. MHC-I structures and corresponding PDB IDs used for docking

| MHC-I allele | PDB ID |
| --- | --- |
| HLA-A*01:01 | 4NQV |
| HLA-A*02:06 | 3OXR |
| HLA-B*07:02 | 5EO1 |
| HLA-B*08:01 | 4QRP |
| HLA-B*15:01 | 1XR9 |
| HLA-B*35:01 | 1A9E |
| HLA-B*44:02 | 3KPL |
| HLA-B*51:01 | 1E28 |
| HLA-B*53:01 | 1A1N |
| HLA-B*57:01 | 3WUW |
| H2-L^d^ | 6L9M |
| H2-K^b^ | 6JQ3 |
| H2-D^b^ | 1JUF |

Supplementary Table 5. Detailed molecular docking results.

| **Genotypes** | **Sequence** | **Best docking score** | **Number of favorable poses** |
| --- | --- | --- | --- |
| HLA-A*02:06 | 141 GAFFLYDRI | -283.564 | 6 |
| HLA-A*01:01 | 141 GAFFLYDRI | -247.218 | 5 |
| HLA-B*07:02 | 141 GAFFLYDRI | -242.774 | 5 |
| HLA-B*35:01 | 141 GAFFLYDRI | -256.707 | 5 |
| HLA-B*51:01 | 141 GAFFLYDRI | -260.498 | 5 |
| HLA-B*53:01 | 141 GAFFLYDRI | -260.524 | 5 |
| HLA-B*08:01 | 141 GAFFLYDRI | -244.009 | 5 |
| HLA-B*15:01 | 141 GAFFLYDRI | -241.075 | 5 |
| HLA-B*44:02 | 141 GAFFLYDRI | -224.402 | 4 |
| H2-L^d^ | 141 GAFFLYDRI | -324.05 | 4 |
| H2-K^b^ | 141 GAFFLYDRI | -222.916 | 5 |
| H2-D^b^ | 141 GAFFLYDRI | -256.482 | 4 |
| HLA-B*57:01 | 141 GAFFLYDRI | -272.969 | 6 |
| HLA-A*02:06 | 440 REGDMFPFL | -251.175 | 5 |
| HLA-A*01:01 | 440 REGDMFPFL | -251.266 | 4 |
| HLA-B*07:02 | 440 REGDMFPFL | -204.98 | 6 |
| HLA-B*35:01 | 440 REGDMFPFL | -227.893 | 5 |
| HLA-B*51:01 | 440 REGDMFPFL | -247.193 | 4 |
| HLA-B*53:01 | 440 REGDMFPFL | -249.162 | 4 |
| HLA-B*08:01 | 440 REGDMFPFL | -217.312 | 6 |
| HLA-B*15:01 | 440 REGDMFPFL | -208.074 | 7 |
| HLA-B*44:02 | 440 REGDMFPFL | -214.447 | 3 |
| H2-L^d^ | 440 REGDMFPFL | -254.834 | 4 |
| H2-K^b^ | 440 REGDMFPFL | -215.515 | 6 |
| H2-D^b^ | 440 REGDMFPFL | -208.67 | 5 |
| HLA-B*57:01 | 440 REGDMFPFL | -231.81 | 7 |
| HLA-A*02:06 | 443 DMFPFLDGL | -225.756 | 5 |
| HLA-A*01:01 | 443 DMFPFLDGL | -249.414 | 5 |
| HLA-B*07:02 | 443 DMFPFLDGL | -217.976 | 5 |
| HLA-B*35:01 | 443 DMFPFLDGL | -222.967 | 4 |
| HLA-B*51:01 | 443 DMFPFLDGL | -229.731 | 4 |
| HLA-B*53:01 | 443 DMFPFLDGL | -251.02 | 5 |
| HLA-B*08:01 | 443 DMFPFLDGL | -213.624 | 6 |
| HLA-B*15:01 | 443 DMFPFLDGL | -211.577 | 5 |
| HLA-B*44:02 | 443 DMFPFLDGL | -193.997 | 5 |
| H2-L^d^ | 443 DMFPFLDGL | -236.954 | 6 |
| H2-K^b^ | 443 DMFPFLDGL | -207.957 | 6 |
| H2-D^b^ | 443 DMFPFLDGL | -197.309 | 6 |
| HLA-B*57:01 | 443 DMFPFLDGL | -227.408 | 6 |
| HLA-A*02:06 | 508 NENDCDAEL | -144.175 | 5 |
| HLA-A*01:01 | 508 NENDCDAEL | -163.189 | 5 |
| HLA-B*07:02 | 508 NENDCDAEL | -124.606 | 4 |
| HLA-B*35:01 | 508 NENDCDAEL | -167.949 | 6 |
| HLA-B*51:01 | 508 NENDCDAEL | -138.353 | 5 |
| HLA-B*53:01 | 508 NENDCDAEL | -156.746 | 3 |
| HLA-B*08:01 | 508 NENDCDAEL | -132.361 | 5 |
| HLA-B*15:01 | 508 NENDCDAEL | -145.23 | 5 |
| HLA-B*44:02 | 508 NENDCDAEL | -135.148 | 5 |
| H2-L^d^ | 508 NENDCDAEL | -154.877 | 5 |
| H2-K^b^ | 508 NENDCDAEL | -127.574 | 6 |
| H2-D^b^ | 508 NENDCDAEL | -148.124 | 5 |
| HLA-B*57:01 | 508 NENDCDAEL | -145.806 | 6 |
| HLA-A*02:06 | 528 AGLSWIPFF | -271.817 | 4 |
| HLA-A*01:01 | 528 AGLSWIPFF | -258.457 | 6 |
| HLA-B*07:02 | 528 AGLSWIPFF | -218.942 | 5 |
| HLA-B*35:01 | 528 AGLSWIPFF | -261.059 | 6 |
| HLA-B*51:01 | 528 AGLSWIPFF | -276.7 | 5 |
| HLA-B*53:01 | 528 AGLSWIPFF | -265.326 | 4 |
| HLA-B*08:01 | 528 AGLSWIPFF | -269.246 | 3 |
| HLA-B*15:01 | 528 AGLSWIPFF | -260.777 | 5 |
| HLA-B*44:02 | 528 AGLSWIPFF | -228.169 | 5 |
| H2-L^d^ | 528 AGLSWIPFF | -279.274 | 5 |
| H2-K^b^ | 528 AGLSWIPFF | -245.502 | 4 |
| H2-D^b^ | 528 AGLSWIPFF | -253.569 | 5 |
| HLA-B*57:01 | 528 AGLSWIPFF | -286.226 | 6 |
| HLA-A*02:06 | 551 NQNNLVCRL | -204.604 | 5 |
| HLA-A*01:01 | 551 NQNNLVCRL | -208.516 | 5 |
| HLA-B*07:02 | 551 NQNNLVCRL | -182.492 | 5 |
| HLA-B*35:01 | 551 NQNNLVCRL | -220.219 | 3 |
| HLA-B*51:01 | 551 NQNNLVCRL | -206.118 | 4 |
| HLA-B*53:01 | 551 NQNNLVCRL | -224.999 | 6 |
| HLA-B*08:01 | 551 NQNNLVCRL | -198.758 | 4 |
| HLA-B*15:01 | 551 NQNNLVCRL | -196.198 | 6 |
| HLA-B*44:02 | 551 NQNNLVCRL | -181.729 | 5 |
| H2-L^d^ | 551 NQNNLVCRL | -230.197 | 5 |
| H2-K^b^ | 551 NQNNLVCRL | -193.659 | 4 |
| H2-D^b^ | 551 NQNNLVCRL | -188.85 | 6 |
| HLA-B*57:01 | 551 NQNNLVCRL | -219.245 | 6 |
| HLA-A*02:06 | 636 TGWGLGGKW | -286.736 | 4 |
| HLA-A*01:01 | 636 TGWGLGGKW | -243.115 | 5 |
| HLA-B*07:02 | 636 TGWGLGGKW | -227.57 | 4 |
| HLA-B*35:01 | 636 TGWGLGGKW | -243.167 | 5 |
| HLA-B*51:01 | 636 TGWGLGGKW | -261.858 | 5 |
| HLA-B*53:01 | 636 TGWGLGGKW | -300.905 | 6 |
| HLA-B*08:01 | 636 TGWGLGGKW | -288.396 | 4 |
| HLA-B*15:01 | 636 TGWGLGGKW | -265.799 | 5 |
| HLA-B*44:02 | 636 TGWGLGGKW | -223.355 | 6 |
| H2-L^d^ | 636 TGWGLGGKW | -341.555 | 4 |
| H2-K^b^ | 636 TGWGLGGKW | -229.903 | 5 |
| H2-D^b^ | 636 TGWGLGGKW | -268.546 | 7 |
| HLA-B*57:01 | 636 TGWGLGGKW | -293.329 | 7 |
| HLA-A*02:06 | 647 SDWGVLTNL | -236.313 | 6 |
| HLA-A*01:01 | 647 SDWGVLTNL | -221.626 | 5 |
| HLA-B*07:02 | 647 SDWGVLTNL | -202.662 | 6 |
| HLA-B*35:01 | 647 SDWGVLTNL | -220.323 | 6 |
| HLA-B*51:01 | 647 SDWGVLTNL | -240.985 | 6 |
| HLA-B*53:01 | 647 SDWGVLTNL | -247.771 | 4 |
| HLA-B*08:01 | 647 SDWGVLTNL | -220.508 | 6 |
| HLA-B*15:01 | 647 SDWGVLTNL | -210.673 | 7 |
| HLA-B*44:02 | 647 SDWGVLTNL | -187.501 | 5 |
| H2-L^d^ | 647 SDWGVLTNL | -242 | 7 |
| H2-K^b^ | 647 SDWGVLTNL | -222.502 | 4 |
| H2-D^b^ | 647 SDWGVLTNL | -202.697 | 5 |
| HLA-B*57:01 | 647 SDWGVLTNL | -230.944 | 7 |

Supplementary Table 6. Allergenicity predictions

| **Position** | **Sequence** | **Prediction** |
| --- | --- | --- |
| 13 | IQGVKTLPI | Allergen |
| 29 | QPQNVDSVC | Allergen |
| 53 | TLSGQKVAD | Allergen |
| 55 | SGQKVADSP | Allergen |
| 56 | GQKVADSPL | Allergen |
| 57 | QKVADSPLE | Allergen |
| 58 | KVADSPLEA | Allergen |
| 60 | ADSPLEASK | Allergen |
| 74 | AGVPPKNVE | Allergen |
| 78 | PKNVEYTEG | Allergen |
| 81 | VEYTEGEEA | Allergen |
| 82 | EYTEGEEAK | Allergen |
| 83 | YTEGEEAKT | Allergen |
| 84 | TEGEEAKTC | Allergen |
| 85 | EGEEAKTCY | Allergen |
| 86 | GEEAKTCYN | Allergen |
| 95 | ISVTDPSGK | Allergen |
| 122 | IHHIQGQNP | Allergen |
| 123 | HHIQGQNPH | Allergen |
| 124 | HIQGQNPHA | Allergen |
| 125 | IQGQNPHAQ | Allergen |
| 127 | GQNPHAQGI | Allergen |
| 128 | QNPHAQGIA | Allergen |
| 130 | PHAQGIALH | Allergen |
| 158 | KVFTEGNIA | Allergen |
| 203 | GTQTNDTGC | Allergen |
| 204 | TQTNDTGCF | Allergen |
| 205 | QTNDTGCFG | Allergen |
| 206 | TNDTGCFGT | Allergen |
| 224 | QTCAPSKKP | Allergen |
| 225 | TCAPSKKPL | Allergen |
| 226 | CAPSKKPLP | Allergen |
| 228 | PSKKPLPLP | Allergen |
| 230 | KKPLPLPTA | Allergen |
| 231 | KPLPLPTAH | Allergen |
| 232 | PLPLPTAHP | Allergen |
| 233 | LPLPTAHPE | Allergen |
| 234 | PLPTAHPEV | Allergen |
| 235 | LPTAHPEVK | Allergen |
| 236 | PTAHPEVKL | Allergen |
| 237 | TAHPEVKLT | Allergen |
| 238 | AHPEVKLTS | Allergen |
| 249 | TDATKLNTT | Allergen |
| 250 | DATKLNTTD | Allergen |
| 251 | ATKLNTTDP | Allergen |
| 269 | SGSGSGEQE | Allergen |
| 270 | GSGSGEQEP | Allergen |
| 271 | SGSGEQEPY | Allergen |
| 272 | GSGEQEPYT | Allergen |
| 273 | SGEQEPYTT | Allergen |
| 274 | GEQEPYTTS | Allergen |
| 277 | EPYTTSDAA | Allergen |
| 279 | YTTSDAATK | Allergen |
| 280 | TTSDAATKQ | Allergen |
| 281 | TSDAATKQG | Allergen |
| 282 | SDAATKQGL | Allergen |
| 283 | DAATKQGLS | Allergen |
| 295 | PPTPSPQPS | Allergen |
| 296 | PTPSPQPST | Allergen |
| 297 | TPSPQPSTP | Allergen |
| 298 | PSPQPSTPQ | Allergen |
| 299 | SPQPSTPQQ | Allergen |
| 300 | PQPSTPQQG | Allergen |
| 301 | QPSTPQQGG | Allergen |
| 302 | PSTPQQGGN | Allergen |
| 304 | TPQQGGNNT | Allergen |
| 305 | PQQGGNNTN | Allergen |
| 314 | HSQGVVTEP | Allergen |
| 315 | SQGVVTEPG | Allergen |
| 316 | QGVVTEPGK | Allergen |
| 317 | GVVTEPGKT | Allergen |
| 318 | VVTEPGKTN | Allergen |
| 319 | VTEPGKTNT | Allergen |
| 320 | TEPGKTNTT | Allergen |
| 321 | EPGKTNTTA | Allergen |
| 322 | PGKTNTTAQ | Allergen |
| 323 | GKTNTTAQP | Allergen |
| 351 | LSTPSVPIQ | Allergen |
| 386 | PTENPTTAK | Allergen |
| 402 | PTTTVPNTT | Allergen |
| 454 | APIDFDPVP | Allergen |
| 495 | FPKVNENTA | Allergen |
| 496 | PKVNENTAH | Allergen |
| 506 | GENENDCDA | Allergen |
| 507 | ENENDCDAE | Allergen |
| 508 | NENDCDAEL | Allergen |
| 509 | ENDCDAELR | Allergen |
| 531 | SWIPFFGPG | Allergen |
| 532 | WIPFFGPGI | Allergen |
| 533 | IPFFGPGIE | Allergen |
| 534 | PFFGPGIEG | Allergen |
| 557 | CRLRRLANQ | Allergen |
| 599 | GGTCKVLGP | Allergen |
| 602 | CKVLGPDCC | Allergen |
| 603 | KVLGPDCCI | Allergen |
| 606 | GPDCCIGIE | Allergen |
| 607 | PDCCIGIED | Allergen |
| 631 | EQKEGTGWG | Allergen |

Supplementary Table 7. Toxicity predictions.

| Seq_601 | TCKVLGPDC | Toxin |
| --- | --- | --- |
| Seq_602 | CKVLGPDCC | Toxin |
| Seq_603 | KVLGPDCCI | Toxin |
| Seq_604 | VLGPDCCIG | Toxin |
| Seq_605 | LGPDCCIGI | Toxin |
| Seq_606 | GPDCCIGIE | Toxin |
| Seq_607 | PDCCIGIED | Toxin |
| Seq_608 | DCCIGIEDL | Toxin |
| Seq_609 | CCIGIEDLS | Toxin |
| Seq_670 | SCICRIFTK | Toxin |
